# Balancing disturbance risk and ecosystem service provisioning in Swiss mountain forests: an increasing challenge under climate change

**DOI:** 10.1007/s10113-022-02015-w

**Published:** 2023-01-23

**Authors:** Timothy Thrippleton, Christian Temperli, Frank Krumm, Reinhard Mey, Jürgen Zell, Sophie Stroheker, Martin M. Gossner, Peter Bebi, Esther Thürig, Janine Schweier

**Affiliations:** 1 Sustainable Forestry, Forest Resources and Management, WSL Birmensdorf, Birmensdorf, Switzerland; 2 Scientific Service National Forest Inventory (LFI), WSL Birmensdorf, Birmensdorf, Switzerland; 3 Mountain Ecosystems, Alpine Environment and Natural Hazards, SLF Davos, Davos, Switzerland; 4 Forest Resources and Management, Resource Analysis, WSL Birmensdorf, Birmensdorf, Switzerland; 5 Swiss Forest Protection, Forest Health and Biotic Interactions, WSL Birmensdorf, Birmensdorf, Switzerland; 6 Forest Entomology, Forest Health and Biotic Interactions, WSL Birmensdorf, Birmensdorf, Switzerland

**Keywords:** Multi-criteria decision support system, Risk predisposition assessment system, Landscape scale, Forest management, *Ips typographus*, Windthrow

## Abstract

Climate change severely affects mountain forests and their ecosystem services, e.g., by altering disturbance regimes. Increasing timber harvest (INC) via a close-to-nature forestry may offer a mitigation strategy to reduce disturbance predisposition. However, little is known about the efficiency of this strategy at the scale of forest enterprises and potential trade-offs with biodiversity and ecosystem services (BES). We applied a decision support system which accounts for disturbance predisposition and BES indicators to evaluate the effect of different harvest intensities and climate change scenarios on windthrow and bark beetle predisposition in a mountain forest enterprise in Switzerland. Simulations were carried out from 2010 to 2100 under historic climate and climate change scenarios (RCP4.5, RCP8.5). In terms of BES, biodiversity (structural and tree species diversity, deadwood amount) as well as timber production, recreation (visual attractiveness), carbon sequestration, and protection against gravitational hazards (rockfall, avalanche and landslides) were assessed. The INC strategy reduced disturbance predisposition to windthrow and bark beetles. However, the mitigation potential for bark beetle disturbance was relatively small (− 2.4%) compared to the opposite effect of climate change (+ 14% for RCP8.5). Besides, the INC strategy increased the share of broadleaved species and resulted in a synergy with recreation and timber production, and a trade-off with carbon sequestration and protection function. Our approach emphasized the disproportionally higher disturbance predisposition under the RCP8.5 climate change scenario, which may threaten currently unaffected mountain forests. Decision support systems accounting for climate change, disturbance predisposition, and BES can help coping with such complex planning situations.

**Supplementary Information:**

The online version contains supplementary material available at 10.1007/s10113-022-02015-w.

## Introduction

Global change impacts due to changing climate and disturbance regimes affect mountain regions more than other biogeographic regions (Price et al. 2011) and are expected to further intensify in the future (Seidl et al. 2017). These changes are particularly relevant, because mountain forests provide a wide range of ecosystem services to society (Mina et al. 2017; Pardos et al. 2017), such as recreation, timber supply, and carbon sequestration, and are important for biodiversity (Krumm et al. 2020). Moreover, the protection of settlements and infrastructure against gravitational hazards is a forest ecosystem service of high importance for mountain regions (Brang et al. 2006; Bebi et al. 2016). In particular, the intensification of large-scale disturbances poses a threat for the continuous maintenance of the protection function, as well as for forest carbon sequestration, recreation, and timber supply, but may also provide opportunities for forest biodiversity by creating stand structural and landscape heterogeneity (Thom and Seidl 2016).

Climate change affects mountain forests in a complex and site-specific way (Bebi et al. 2016). The differentiated effects of climate change are most pronounced along elevational gradients, ranging from positive effects on tree growth at higher, currently temperature-limited elevations, to negative effects at lower, water-limited elevations (Lindner et al. 2010; Elkin et al. 2013). Furthermore, climate change also alters the frequency, magnitude, and intensity of disturbances (Seidl et al. 2017). For mountain regions in Central Europe, this may result in an increased likelihood of strong wind events (Bolte et al. 2009; Seidl et al. 2017). Moreover, European mountain forests dominated by Norway spruce (*Picea abies* (L.) Karst.) are particularly threatened by the spread of the spruce bark beetle (*Ips typographus* (L.)) (Lindner et al. 2010; Hlásny et al. 2021a). This development is on the one side driven directly by climate change due to an increasing drought-related susceptibility of spruce and increasing generation numbers of bark beetle under warming conditions (Wermelinger and Seifert 1998). On the other side, it is strongly dependent on local forest structures determining the availability of suitable beetle breeding material, preferentially old spruce trees of large diameter or lying deadwood from previous windthrows (Netherer and Nopp-Mayr 2005; Seidl et al. 2007a; Temperli et al. 2013). Assessing climate change effects on mountain forests is thus complex and depends on the interplay of regional environmental and local, stand-scale drivers of abiotic and biotic disturbance regimes (Sommerfeld et al. 2021).

Accounting for climate change and disturbance risk as well as for biodiversity and ecosystem services (BES) is a particularly complex challenge for forest management in mountain regions (Bont et al. 2019; Schweier et al. 2019). As a guideline, the principles of adaptive and “climate-smart” forestry have been developed, which aim at enhancing forest resistance and resilience against climate change and disturbance impacts (Bolte et al. 2009; Jactel et al. 2009; Nabuurs et al. 2017; Mathys et al. 2021). To reduce forests’ predisposition to windthrow and bark beetle disturbance risk in the long term, it is important to adapt susceptible forests accordingly. For mountain forests with a protective function, increasing timber harvest via a close-to-nature forestry may offer an approach to mitigate disturbance predisposition. This can be obtained by small-scale selective cuts aiming at (1) reducing spruce abundance and increasing structural and tree species diversity via fostering of natural regeneration (Larsen et al., 2022) and (2) removing tall, old trees which have a higher wind loading (Hale et al. 2012), and are preferred breeding habitats for bark beetles (Seidl et al. 2007a). However, the effectiveness of increasing harvest intensity for disturbance mitigation is controversially discussed, and may be influenced by climate change (Brang et al. 2006; Zimová et al. 2020). Moreover, increasing timber harvest may result in trade-offs with biodiversity and ecosystem service provisioning, e.g., carbon storage in the forest ecosystem (Stadelmann et al. 2021). Forest management planning therefore needs to carefully assess the effectiveness of recommended mitigation strategies at the forest enterprise level and account for trade-offs and synergies with biodiversity and ecologically, socially, and economically relevant ecosystem services (Pardos et al. 2017; Hlásny et al. 2021a).

To cope with the complex planning situation at the scale of a forest enterprise (in Central Europe typically between 100 and 1000 s ha, which corresponds to the landscape scale, Keane et al. 2015), decision support systems (DSS) have been developed, that provide forest enterprise managers with a comprehensive assessment of the consequences of management approaches (Vacik and Lexer 2014; Blattert et al. 2018). While the first generations of DSS focused primarily on wood production, newer systems often consider a range of BES indicators (Blattert et al. 2017). Furthermore, DSS are increasingly coupled to multi-criteria decision analyses (MCDA), which allow the integration of multiple, often conflicting criteria and stakeholder preferences to address complex decision problems in a structured and transparent way (Uhde et al. 2015). These multi-criteria decision support systems (MCDSS) often provide a measure of multifunctionality, e.g., in the form of an overall utility (Kangas et al. 2015). Based on utility theory, the overall utility summarizes the performance of individual BES indicators and allows to compare alternative scenarios (Blattert et al., 2018). Although many MCDSS feature a wide portfolio of BES, they typically lack an evaluation of disturbance risk, particularly in a dynamic, climate-sensitive way, that considers also the link between disturbances (Seidl et al. 2017). Suitable assessment systems for windthrow and bark beetle disturbances have been developed in the past (Netherer and Nopp-Mayr 2005) and applied at various scales outside MCDSS (Temperli et al. 2013; Jakoby et al. 2019; Puhlmann and Hallas 2020). An integration of disturbance predisposition into a MCDSS at the forest enterprise level is therefore an important step to improve decision support for forest management (Stritih et al. 2021), and allows to assess enterprise-specific management strategies for a continuous and well-balanced ecosystem service provisioning (Hlásny et al. 2021a).

Here, we employed a MCDSS for forest enterprises (Thrippleton et al. 2021), which considers biodiversity and ecosystem services in terms of provisioning (timber production), regulatory (carbon sequestration, protection against rockfall, and avalanches), and cultural (recreation) services, and extended it by integrating windthrow and bark beetle disturbance predisposition (Netherer and Nopp-Mayr 2005; Temperli et al. 2020). The aim of the study was to evaluate the effect of different harvest intensities on disturbance predisposition and assess potential trade-offs with BES under a set of climate change scenarios. We focused particularly on the long-term development under changing mean climatic conditions to assess general trends, rather than the impacts of extreme climatic events. Since mountain forest regions are future hot-spots of climate change and disturbance impacts (Elkin et al. 2013; Albrich et al. 2020), the MCDSS was applied to the high-elevation forest landscape around Davos in Switzerland (Schumacher et al. 2004). The case study represents a typical high-elevation (> 1500 m a.s.l.) spruce-dominated mountain forest enterprise. While the area is currently largely unaffected by bark beetle outbreaks, climate change is likely to alter the situation, leading to significant warming (and to a lesser degree drought) and increasing disturbance frequency (Elkin et al. 2013). We therefore asked the following research questions:

1. Can an increase in harvest intensity mitigate the long-term disturbance predisposition to windthrow and bark beetle attacks under present climate conditions?
2. Does the efficiency of disturbance mitigation decrease under changing climatic conditions in the future?
3. Which trade-offs and synergies occur between disturbance mitigation and BES, and how does disturbance mitigation affect the multifunctionality (i.e., overall utility)?

## Materials and methods

### Case study area

**Fig. 1:**
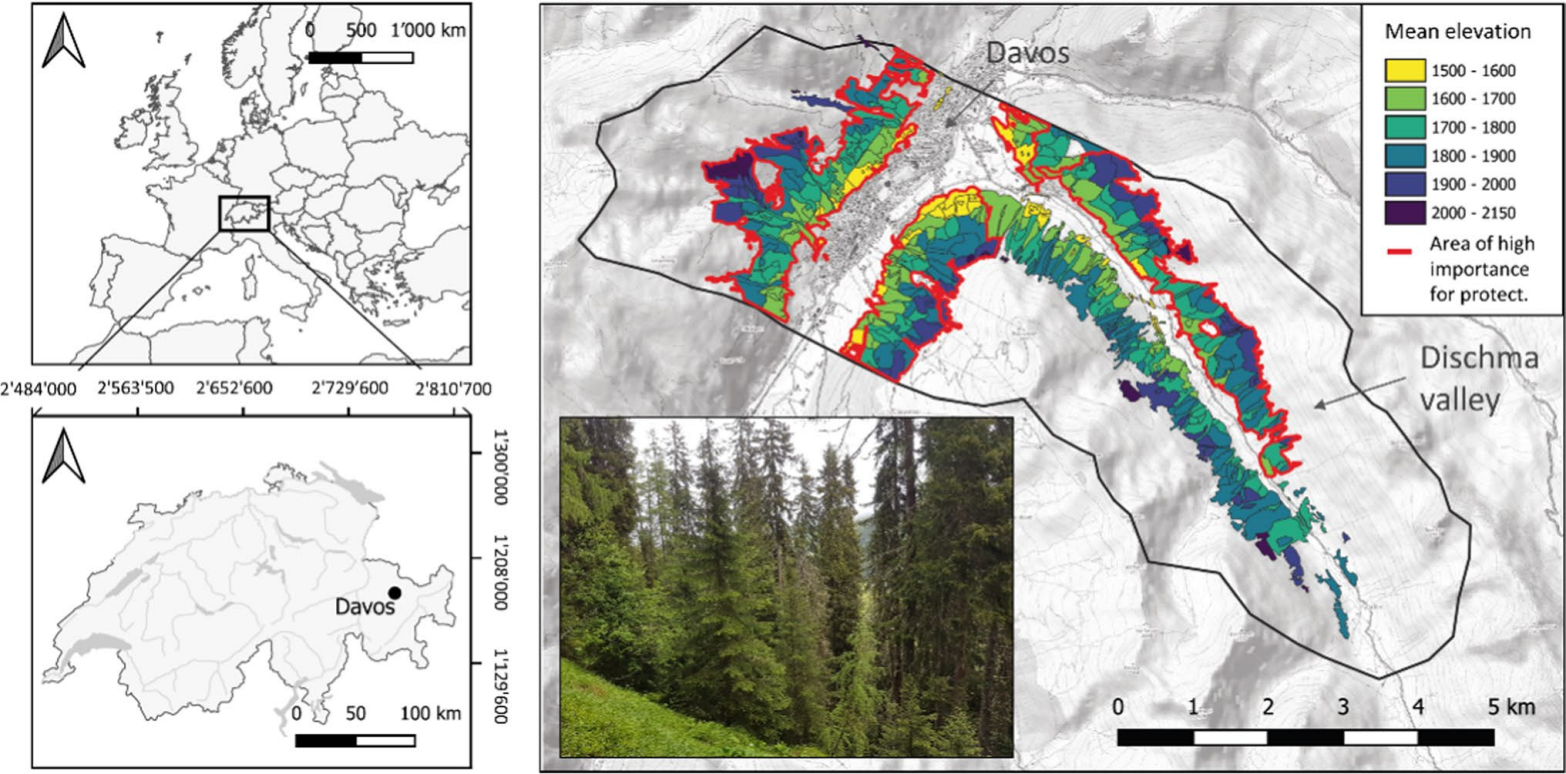
Location and stand map of case study area Davos and Dischma valley in Switzerland. Red lines mark stands of high protective importance; colors of the stands indicate mean elevation (m a.s.l.) (background map © open topo map)

The case study area is located in Grisons, Switzerland, and covers the forests surrounding the city of Davos (46°48′37″N, 9°50′16″E) and the adjacent Dischma valley (Fig. 1). It comprises a landscape with an area of 1350 ha (in total 565 individual forest stands), with an average standing volume of 225 m^3^ ha^−1^, dominated by stands in the sawtimber stage (i.e., average DBH > 25 cm) (Stadler et al. 2015). Timber harvest is mostly carried out via helicopter and cable yarding due to steep terrain conditions, and focuses predominantly on forests with protection function, where forest management is generally economically unprofitable and hence subsidized (Stadler et al. 2015). The environmental setting at the case study area is typical for high-elevation Alpine conditions, with a mean annual temperature of 3.5 °C (mean summer temperature of 11.1 °C and mean winter temperature of − 4.3 °C) and annual precipitation of 1020 mm (recorded at climate station Davos-Platz for period 1981–2010, Petter et al. 2020). Soils in the study area are predominantly loamy sands and sandy loams, with generally higher water holding capacity at the valley bottom and shallow soils of lower water holding capacity at steeper slopes (Petter et al. 2020). The forest extends from the valley bottom (1560 m a.s.l.) to the upper treeline (ca. 2250 m. a.s.l.). It is dominated by Norway Spruce (*Picea abies* (L.) Karst.), as well as by European Larch (*Larix decidua* Mill.) and Swiss stone pine (*Pinus cembra* L.) at higher elevations or in avalanche tracks. Broadleaves (e.g., green alder, birch, and rowan) are admixed when the light conditions are favorable.

### Decision support system

**Fig. 2:**
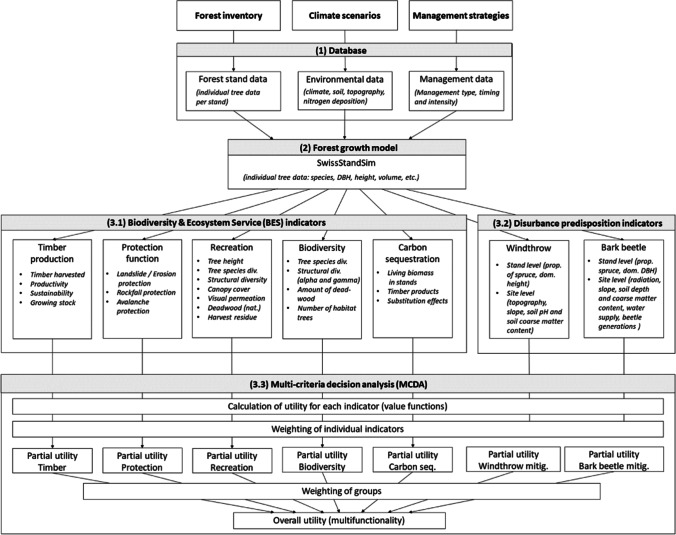
Conceptual diagram of the MCDSS structure (modified from Thrippleton et al. 2021), comprising three main components (1: database, 2: forest growth model, 3: indicator framework and multi-criteria decision analysis) and considering biodiversity and ecosystem services (3.1) and disturbance predisposition indicators (3.2)

A MCDSS developed for forest enterprises in Switzerland (Thrippleton et al. 2021) was used to study the effect of harvest intensity on disturbance predisposition and BES. The system consists of three core components (Fig. 2), a database (defining initial stand conditions, environmental conditions, and management), a forest growth model (SwissStandSim, Zell et al. 2020), and a multi-criteria decision analysis component based on Blattert et al. (2018). For the present study, the existing MCDSS indicator system was extended to include also disturbance predisposition indicators for windthrow and bark beetle (Netherer and Nopp-Mayr 2005; Temperli et al. 2020) (Fig. 2). The system allows to calculate the predisposition of stands to disturbances without explicitly simulating the occurrence of disturbance events.

### Database

The database for the MCDSS contains basic information for the application of the forest growth model regarding (1) environmental conditions, (2) stand structure, and (3) management (i.e., type and intensity of timber harvest) for each stand (Zell et al. 2020). To describe environmental conditions, data for topography (elevation, aspect, slope) were derived from the digital elevation map of Switzerland (© Swisstopo 2010), for soil (soil depth, texture, water permeability, water retention capacity, and nutrient availability) from the MAB project (Wildi and Ewald 1986) and for nitrogen deposition from the Swiss nitrogen deposition map (FOEN 2015). Climate data (historic and future climate scenarios) for the location of Davos was extracted from the Swiss-wide climate datasets of Brunner et al. (2019).

Stand-level individual tree datasets were created using forest inventory data of the canton of Grisons for the region of Davos (AWN (Cantonal Office for Forest and Natural Hazards of Graubünden), Cantonal Forest Inventory, 2018a). The sampling method of this inventory is based on the Swiss National forest inventory (Fischer and Traub 2019). The 500 m × 500-m sampling grid of the forest inventory of Grisons intersected only with a minority of stands in the Davos study area. To overcome this restriction, we used a “representative stand type” approach, as frequently done in comparable forest modelling studies (Seidl et al. 2007b; Pardos et al. 2017). Based on the forest stand map (Stadler et al. 2015), all stands were assigned to groups of “stand types,” based on (1) tree species composition, (2) developmental stage, and (3) topography (elevation and aspect). For each stand type, complete stand-level datasets were created using the statistical approach of Mey et al. (2021) and assigned to each stand for the forest growth simulation. Further details about the stand initialization is provided in Appendix A1.

### Forest growth model

The empirical, climate-sensitive individual-tree model SwissStandSim (Zell et al. 2020) was employed in the MCDSS to simulate forest development at the stand scale under present and future climatic conditions. SwissStandSim was developed based on a large empirical dataset from the experimental forest growth and yield network in Switzerland (Forrester et al. 2019), see also Appendix A1.0. The dataset features long time series of stand development (up to 112 years) and covered a large environmental gradient from warm-dry conditions in low-elevation stands of South-Western Switzerland to temperature-limited high-elevation stands in Central-Alpine forests (Zell 2018). The model represents harvest and individual tree demography via statistical sub-models. The demographic processes of ingrowth (i.e., regeneration), growth, and mortality are represented by species-specific sub-models for 11 tree species. The sub-models feature effects of individual tree conditions (e.g., diameter at breast height, age), competition (e.g., competition by larger trees), stand conditions (e.g., stand density, species mixture), site conditions (e.g., nitrogen deposition), and climatic conditions. All demographic processes are climate sensitive and consider the influence of annual mean temperature and precipitation sum. For the process of ingrowth (i.e., tree regeneration), changes in mean temperature alter the number of ingrowing trees and the species composition (Mey et al. 2022). Stochasticity is considered via stochastic elements in the harvesting, ingrowth, and mortality sub-models. The temporal resolution of the model is 5 years, which allows to simulate forest development under changing mean climatic conditions, but does not represent the effect of extreme climatic events (e.g., severe drought events). The model performance for tree growth has been cross-validated in a previous study by Zell et al. (2018), showing a high predictive power compared to tree growth models developed from the Swiss national forest inventory (Rohner et al. 2018) (see Appendix A1.0). SwissStandSim has furthermore been evaluated at a high-elevation spruce forest (1500 m a.s.l.), where long observation time series were available and similar conditions to our case study area Davos prevailed. The results of this comparison showed a good prediction of growth and stand-development and deviations in the long term due to an absence of disturbance events in the simulations (Zell et al. 2020). With its strong empirical foundation and suitability for a wide range of environmental conditions in Switzerland, SwissStandSim meets important requirements for a DSS for forest practitioners (Vacik and Lexer 2014; Thrippleton et al. 2021).

### Biodiversity and ecosystem service indicators

For the quantification of biodiversity and ecosystem services (BES), the indicator set developed by Blattert et al. (2017) was used.

For the protection function, the effect of the forest on gravitational hazards by rockfall, avalanche, and landslides wAS calculated using the indicator set of Blattert et al. (2017), originally developed by Cordonnier et al. (2014). These indicators are based on slope conditions and stand characteristics, and are scaled between 0 (low protection) and 1 (very high protection). The rockfall protection index (RPI) was developed on the basis of the model Rockfor^Net^ (Dorren et al. 2004; Berger and Dorren 2007) and measures the energy dissipation ability of the forest stand relative to the dissipating maximal energy of a falling rock (Appendix A1.2.1). The avalanche protection index (API) expresses protection based on the relationship between current stand characteristics and stand characteristics required for an optimal protection. Notably, it was assumed that forest avalanches occur only at slopes of > 28° and elevations > 800 m a.s.l., following Cordonnier et al. (2014) (Appendix A1.2.2). The landslide and erosion protection index (LPI) is based on the extent of forest canopy cover and was developed on the basis of Frehner et al. (2005) (Appendix A1.2.3).

For quantifying sustainable timber provisioning, the amount of timber volume harvested (m^3^ ha^−1^ year^−1^), productivity (annual net volume increment), sustainability of timber use (i.e., the ratio between harvest and productivity), and the growing stock of the stands were considered.

For quantifying biodiversity, tree species diversity (using the Shannon index, Shannon, and Weaver 1949) and structural diversity (using the Post Hoc index, Staudhammer and LeMay 2001) were considered in terms of alpha diversity (representing diversity within each stand) and gamma diversity (representing diversity within the forest enterprise, i.e., at the landscape scale). The amount of deadwood (accounting for deadwood accumulation by natural mortality and harvest residue, as well as decomposition) and the number of habitat trees (i.e., trees bearing microhabitats (Larrieu et al. 2018), here assumed as large, old trees with a diameter of > 70 cm) were furthermore considered, due to their importance for various taxonomic groups (Gossner et al. 2013).

The recreation value was calculated based on structural attributes of each stand, which have been linked to visual attractiveness in a large-scale evaluation study by Edwards et al. (2012). The indicators comprise the size of the largest trees (m), variation in tree size (post hoc index), extent of canopy cover (i.e., percentage of ground covered by canopy), visual permeation through stand (expressed via the stand density index, Daniel and Sterba 1980), variation in tree species (Shannon index), residues from harvest and thinning, and deadwood from natural mortality (deadwood volume in m^3^ ha^−1^).

For quantifying carbon sequestration, the carbon stored in the living biomass (above and belowground) within the stand (“in situ sequestration”), the carbon stored in harvested wood products (“ex situ sequestration”), and substitution effects were considered using a modified approach of Blattert et al. (2017), as described in Thrippleton et al. (2021). Carbon stored in the living biomass was calculated using species-, region-, and elevation-specific allometric equations from the Swiss National Forest Inventory (Didion et al. 2019). Deadwood originating from natural mortality and harvest residues, as well as its temperature-dependent decomposition, was considered (Blattert et al. 2018; Thrippleton et al. 2021). Harvested wood products were considered according to product classes and their longevity, i.e., sawnwood, wood-based panels, paper, and paperboard (IPCC 2014). Furthermore, the substitution effect of using wood as a construction material and for energy production instead of concrete or fossil fuels were considered, based on Taverna et al. (2007).

A detailed description of the calculation of all biodiversity and ecosystem service indicators is provided in Thrippleton et al. (2021) and appendices therein.

### Disturbance predisposition assessment

The evaluation of disturbance predisposition from windthrows and bark beetles was based on the predisposition assessment system by Netherer and Nopp-Mayr (2005), which was adapted by Temperli et al. (2020) for a dynamic forest modeling framework. The assessment system focuses on the predisposition of stands in a forest enterprise instead of disturbance occurrence since the predisposition status can be influenced by the forest manager via alternative management strategies. The system was developed by Netherer and Nopp-Mayr (2005) as a knowledge-based expert model on the foundation of an extensive literature review and knowledge from experts of entomology, phytopathology, forest protection, forest ecology, silviculture, and wildlife biology. In this approach, a range of predisposing factors for disturbance risk are evaluated, comprising site-related factors (i.e., the environmental conditions at a specific location) and stand-specific factors (i.e., stand structural characteristics that increase the probability of a disturbance). All site- and stand-related factors were integrated based on an expert-defined weighting set reflecting their relative importance for the disturbance risk (see Appendix A1.3). The resulting predisposition indicator expresses the relative predisposition level (ranging from 0 as the lowest level to 1 as the highest level) and can be interpreted as a propensity to be damaged by a disturbance agent.

For windthrow, the predisposition indicators comprise: proportion of spruce and dominant height (stand-related factors), topographic exposure, slope, soil coarse matter content (> 2 mm), and pH of the soil (site-related factors). For bark beetle, the predisposition indicators comprise: proportion of spruce, dominant diameter (stand-related factors), radiation, slope position, soil coarse matter content (> 2 mm, which influences soil water holding capacity), soil depth, water supply (expressed via the drought index by Bugmann and Cramer 1998), and number of beetle generations (site-related factors). Climate change influences the disturbance predisposition hence indirectly via changes in stand-related factors and directly via the site-related factors water supply and number of beetle generations. Furthermore, the risk of windthrows is included as an influencing factor on bark beetle risk since the link between both disturbances is a key factor which needs to be accounted for in assessments (Temperli et al. 2013). The assessment system is considered as a sound representation of disturbance predisposition and is widely used in Central Europe (e.g., Jakoby et al. 2016; Puhlmann and Hallas 2020). The adapted version of Temperli et al. (2020) has been validated using data from storm damages and insect (mostly bark beetle) damages recorded in the national forest inventory of Switzerland (Temperli et al. 2020). For the present study, the factor “number of bark beetle generations” was implemented in the MCDSS as a dynamic temperature-dependent relationship based on results of Jakoby et al. (2016) and Jakoby et al. (2019). Further details about the approach are provided in Appendix A1.3. A detailed description of the calculation of all disturbance predisposition indicators is provided in Temperli et al. (2021) and appendices therein.

### Multi-criteria decision analysis

For the evaluation of multifunctionality, the system features a MCDA (Fig. 2) approach based on the multi-attribute value theory, which allows to compare different management strategies and evaluate their effect on BES (Kangas et al. 2015). The approach furthermore allows stakeholders to express their preferences by assigning weights to criteria. Overall, the MCDA is considered as an important part of DSS, facilitating a transparent and structured decision process (Kangas et al. 2015; Uhde et al. 2015; Schweier et al. 2019). For the MCDA, the simulated BES and disturbance indicators are aggregated into partial and overall utilities (see also Fig. 2) to measure the degree of multifunctionality (Kangas et al. 2015). As a first step, BES indicators are calculated from the simulation results of the forest model. As a second step, the indicator values are converted to a utility scale, which ranges between 0 (lowest utility) and 1 (highest utility). For this conversion, the value functions of Blattert et al. (2017) are used, which are mathematical expressions representing human judgment of supply and benefit of BES (Ananda and Herath 2009). As a next step, the individual indicators of each indicator group and their respective stakeholder-defined weighting factors (λ_a,i_, Table A1.6) are combined into a partial utility of each indicator group. Finally, the partial utilities of all indicator groups are combined into an overall utility (as a measure for multifunctionality), using weighting factors for each indicator group (λ_a_, Table A1.6). For the calculation of partial and overall utilities, an additive function was used (Kangas et al. 2015):

$$\mathrm{overall\; utility}= {\sum }_{\mathrm{a}=1}^{\mathrm{m}}{\lambda }_{a}\left({\sum }_{\mathrm{a},\mathrm{i}=1}^{{\mathrm{n}}_{\mathrm{a}}}{\lambda }_{a,i} {V}_{a,i}\text{ (}{Y}_{a,i}\text{)}\right)$$

with:

$${\sum }_{\mathrm{a},\mathrm{ i}=1}^{{\mathrm{n}}_{\mathrm{a}}}{\lambda }_{a,i}=1 ,\;\mathrm{ for\; all\; a}$$

$${\sum }_{\mathrm{a}=1}^{\mathrm{m}}{\lambda }_{a}=1$$

where V_a,i_ (Y_a,i_) is the normalized utility for each individual indicator (i) per indicator group (a) based on the value function, λ_a_ are weights of each indicator group, λ_a,i_ are weights of each individual indicator per group, *n*_*a*_ the number of individual indicators per group, and *m* the number of groups. A more detailed description of the MCDA approach is provided in Thrippleton et al. (2021).

### Simulation scenarios

Simulations were conducted for three management strategies and four climate scenarios. The investigated management strategies focused on different timber harvest intensities, which represents a typical management alternative in the region (Temperli et al. 2017). Since no stand-specific management descriptions were available, the current management regime was implemented based on an expert assessment of mountain forest management in this region by Bircher (2015), which has also been applied in other studies (Huber et al. 2021). Based on this assessment, harvest intervals of 30 years and a harvest intensity of 35% basal area removal via a close-to-nature forest management (i.e., small-scale selective cuts which focus on the removal of large trees to foster natural regeneration, see also Ott et al. 1997) were assumed for current management (“MED”). Notably, the current management is characterized by an underutilization of timber, leading to an increase in stand volume over time (Stadler et al. 2015). Further details of the management regime (i.e., priority of stands for harvesting) were implemented according to the forest enterprise plan of Davos (Stadler et al. 2015) (see Appendix A1.4). Furthermore, a decreased intensity strategy (DEC, basal area removal of 25%) and an increased intensity strategy (INC, basal area removal of 45%) were investigated, based on the typical range of harvest intensities in Swiss mountain forests (Temperli et al. 2017). For the DEC and INC strategy, the same harvest intervals as for MED were assumed. No site-specific tree species selection or deadwood retention strategies were considered. Furthermore, only natural regeneration was considered (i.e., no planting was simulated) and shifts in tree species composition occurred due to changes in natural regeneration.

Climate scenarios comprised a “historic climate” scenario (climate conditions from the reference period 1981–2010, corresponding to “current climate conditions” at the start of the simulation), and three typical “wet,” “medium,” and “dry” climate change (CC) scenarios for the timespan 2010 to 2100 from Brunner et al. (2019). The CC scenarios comprise three climate model chains covering a range from wet conditions (CC22), to medium (CC7) conditions under a “moderate warming” scenario (RCP4.5) and dry conditions (CC1) under a “high warming” scenario (RCP8.5) (Table A 1.5). The CC scenarios were downscaled using a regional downscaling approach based on quantile mapping (Gudmundsson et al. 2012). Further details about the climate scenarios are provided in Appendix A1.5. Simulations were carried out for all forest stands for the years 2010 to 2100.

### Analysis of disturbance predisposition and BES

The effect of different harvest intensities and climate change on disturbance predisposition was quantified as the magnitude of differences between the simulated scenarios (see recommendation of White et al. 2014). For the effect of harvest intensity, the change of disturbance predisposition relative to the current (“MED”) management intensity was calculated. For the effect of climate change, the change in the disturbance index relative to historic climate conditions (“Hist”) and current management (“MED”) was calculated. Since the focus of the study was on the long-term effect of forest management, the average change of the predisposition index from 2010 to 2100 was quantified. Trade-offs and synergies were analyzed using Spearman’s rank correlation coefficient between the mean partial utilities over the simulation timespan of the different BES groups, including the results of all management strategies and climate change scenarios (see also Thrippleton et al. (2021)). For the identification of the management strategy resulting in the highest multifunctionality, the MCDA was calculated for all BES groups and both disturbance indicators (Fig. 2). The weighting factors for the MCDA were estimated based on descriptions of BES preference from the regional and enterprise-level forest development plan (Stadler et al. 2015; AWN 2018b), giving highest priority to the protection function, intermediate weight to timber production, biodiversity and recreation (visual attractiveness), and a lower weight to carbon sequestration (see also BES prioritization by Cathomen and Vanoni 2020). A detailed overview over the weighting factors is provided in Table A1.6. The MCDSS framework, as well as all calculations and visualizations were conducted with R version 4.0.0 (R Core Team 2020). For the visualization of the trade-offs and synergies via the chord diagram, the R package “circlize” was used (Gu et al. 2014).

## Results

### Effect of harvest intensity on disturbance predisposition under historic climate

**Fig. 3:**
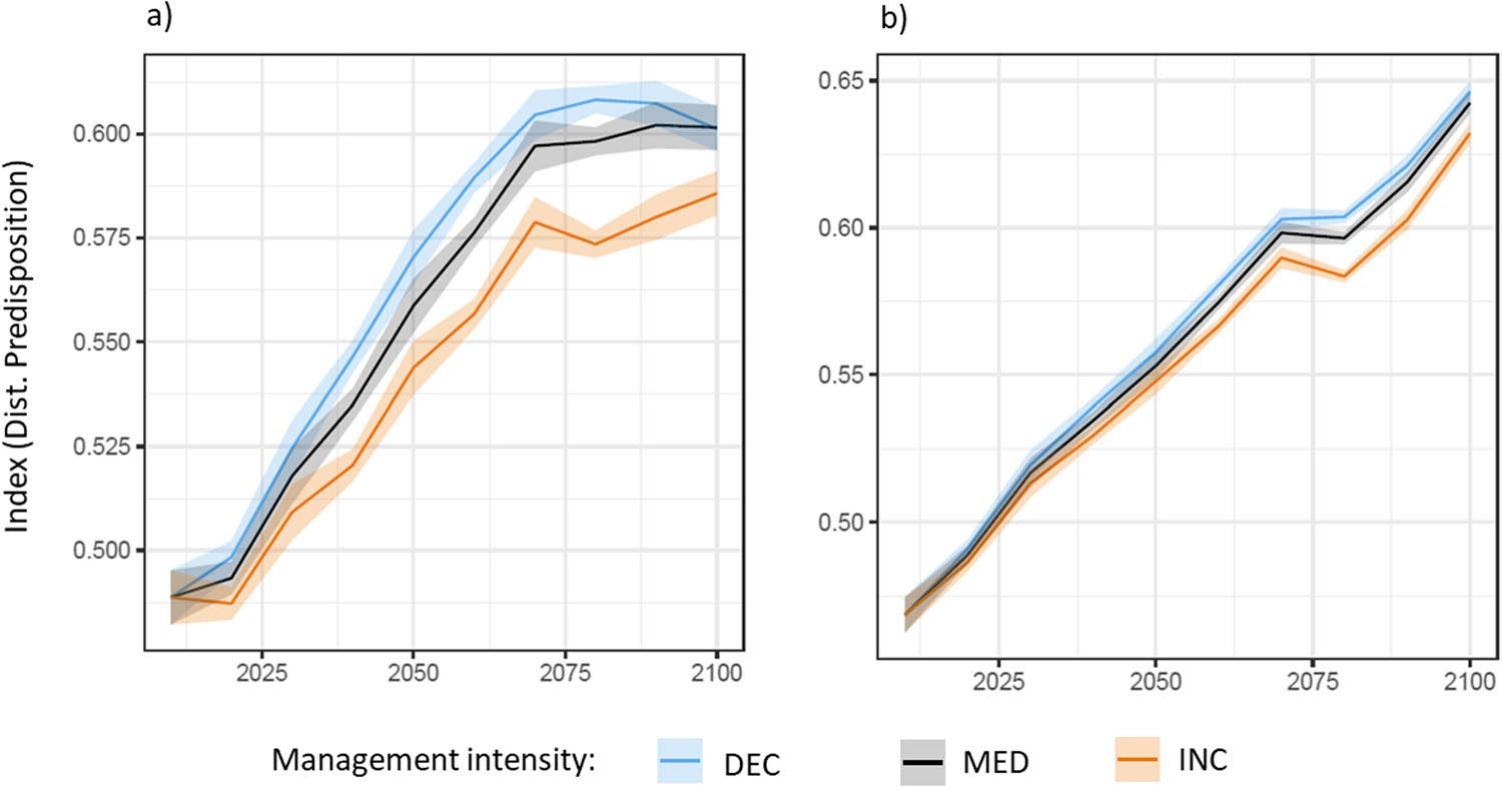
Development of disturbance predisposition for **a** windthrow and **b** bark beetle under three harvest intensities under historic climate conditions (mean and standard error of the mean across all stands of the enterprise)

**Fig. 4:**
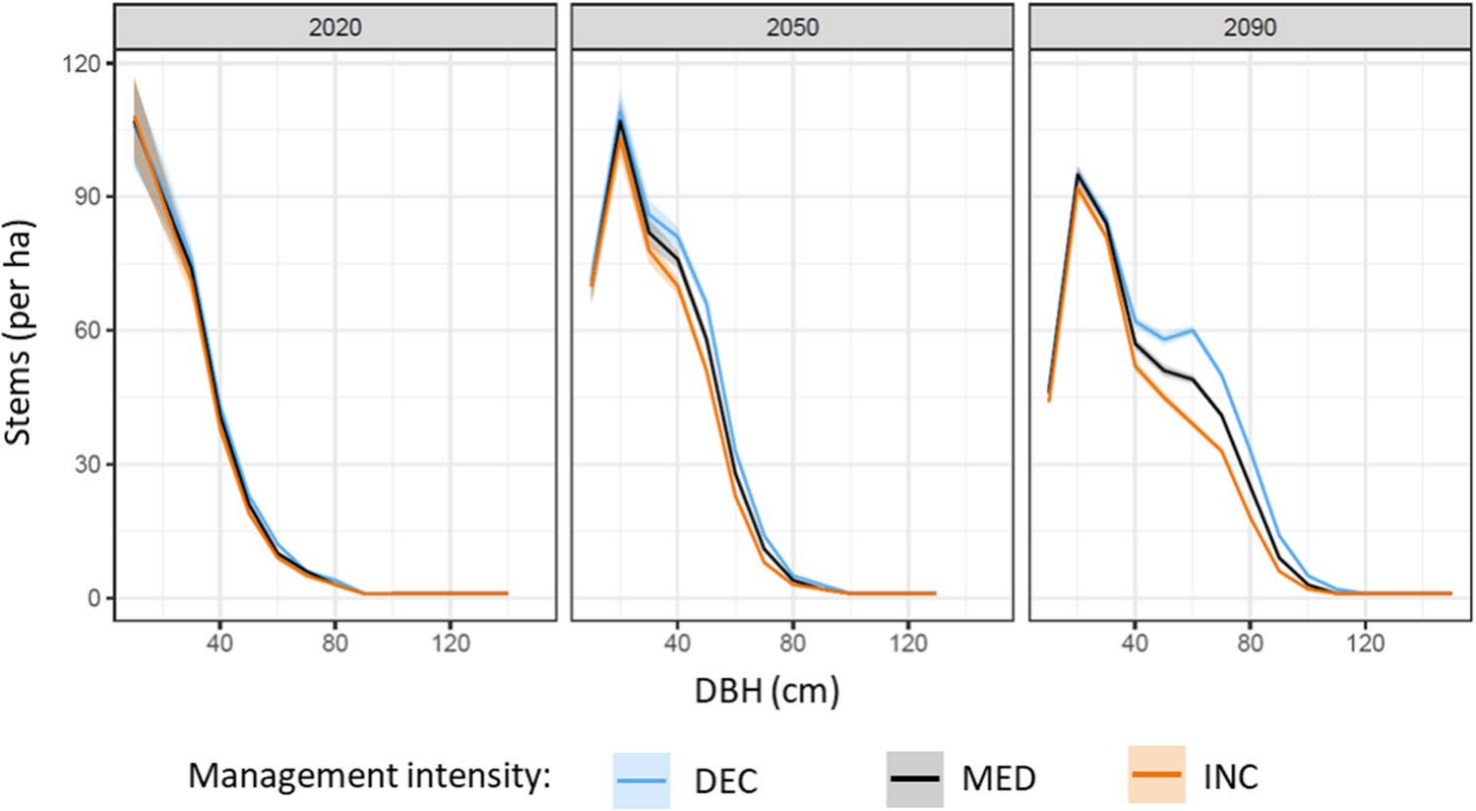
Development of mean diameter distributions over time (mean and standard error of the mean across all stands of the enterprise for years 2020, 2050, and 2090) under three different harvest intensities for historic climate

Under historic climate conditions, the predisposition to both disturbances increased over time under all harvest intensities (average increase of 0.11 for windthrow and 0.17 for bark beetle until 2100), implying that the INC strategy was not sufficient to decrease the overall disturbance predisposition in the long term. In comparison to the current harvest intensity (MED), the INC strategy led to a relative decrease of both disturbance predispositions, while the opposite pattern occurred for the DEC strategy (Fig. 3). The management effect was stronger for predisposition to windthrow (Fig. 3a) than for bark beetle disturbance (Fig. 3b). The overall increase in disturbance predisposition with time (Fig. 3) was related to the increasing number of trees with large diameters across all stands of the forest enterprise (Fig. 4).

### Impact of climate change on disturbance predisposition

**Fig. 5:**
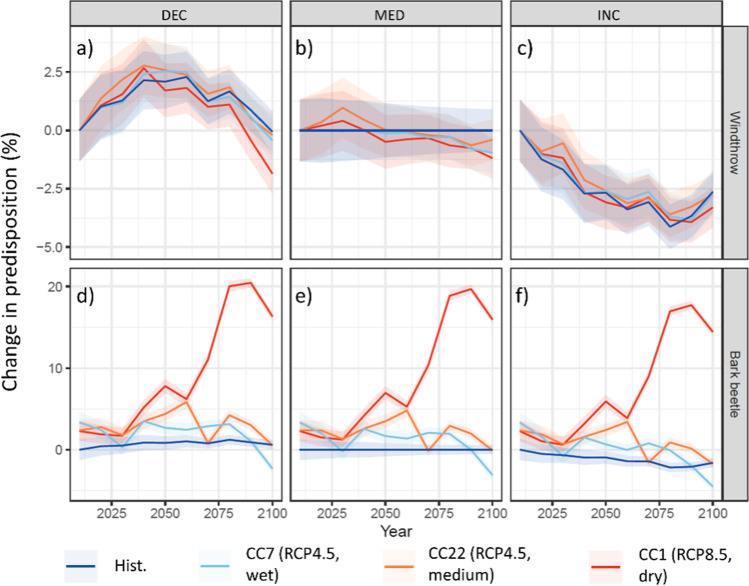
Change in disturbance predisposition relative to current management and historic climate conditions (“Hist” and “MED”) for windthrow and bark beetle under different harvest intensities and climate change scenarios. Note the different scales of the y-axes for windthrow and bark beetle disturbances

The climate change scenarios caused minor differences relative to the historic climate for windthrow and larger differences for bark beetle disturbances (Fig. 5). For windthrow (Fig. 5a–c), the effect of changing harvest intensity on disturbance predisposition was similar under all climate change scenarios, leading to an increased disturbance predisposition under the DEC (mean of + 1.2% for all CC scenarios) and a decreased predisposition under the INC strategy (mean of − 2.4% for all CC scenarios), relative to the MED strategy under historic climate. For bark beetle (Fig. 5d–f), the management strategies had the same general effect on disturbance predisposition, but the overall magnitude was small compared to the impact of climate change. While the “moderate” (RCP4.5) climate scenarios CC7 and CC22 led to a moderate increase (mean of + 0.45% and + 1.9% for MED) in bark beetle disturbance predisposition, the “high impact” (RCP8.5) climate scenario CC1 resulted in a disproportionally stronger increase (mean of + 14% for MED) in predisposition, particularly after the year 2050 (Fig. 5d–f).

These patterns were mainly caused by the changes in the number of bark beetle generations and water supply (i.e., drought stress) for spruce. While the number of bark beetle generations remained on average at one generation per year for historic climate and the CC7 and CC22 scenarios, the warmer conditions under the CC1 scenario led to an increase of up to two generations per year until the end of the twenty-first century (Appendix A2.1a). Similarly, water supply remained at a relatively high level (drought index < 0.3) for the historic climate and the CC7 and CC22 scenarios, whereas it decreased for the CC1 scenario by the mid-twenty-first century (drought index > 0.5, see Appendix A2.1b). Besides, the simulated increase in climate change and harvest intensity led to a decreasing trend in the percentage of spruce until 2100 (up to − 7% for CC1 under the INC strategy), and a rising share of broadleaved trees (up to + 2.5% for CC1 under the INC strategy, Fig. A2.2). The effects of climate change on basal area and deadwood development were more variable and not directly related to warming and drying trends in the climate change scenarios (Fig. A2.3).

### Trade-offs, synergies, and multifunctionality evaluation

**Fig. 6:**
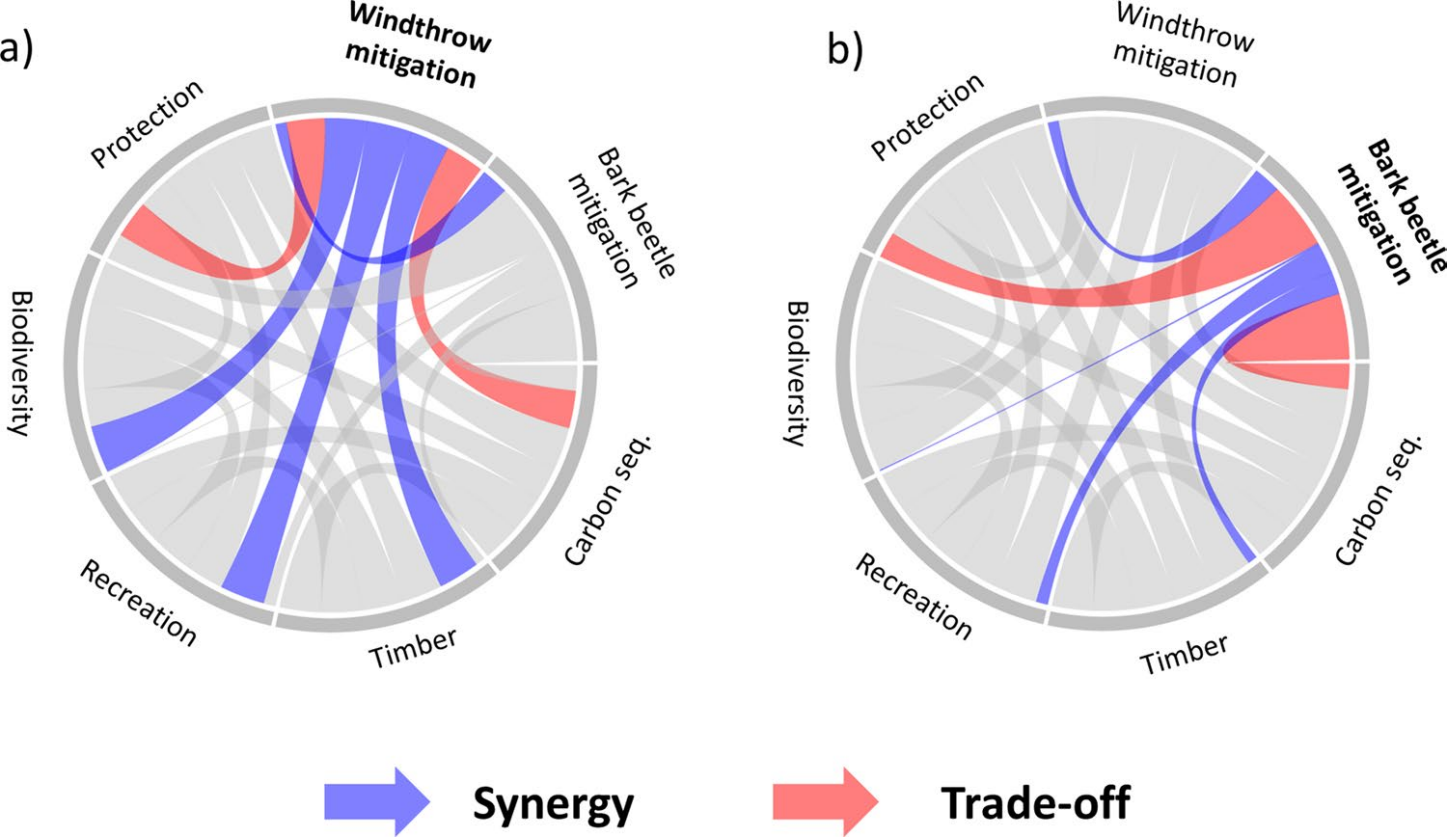
Synergies and trade-offs (i.e., positive and negative relationships, respectively) between **a** windthrow and **b** bark beetle disturbance mitigation (via the INC strategy), biodiversity, and ecosystem service provisioning for the case study area Davos. Note that the size of the connection corresponds to Spearman’s correlation coefficient (see Appendix Fig. 2.4)

Mitigating windthrow and bark beetle disturbance predisposition by increasing harvest intensity (INC strategy) resulted in trade-offs with carbon sequestration and protection function (Fig. 6a, b). The negative effect on carbon sequestration resulted from a relative reduction of carbon stored in living biomass within the stands (Fig. A2.5). The trade-off with protection function was caused by a minor reduction in all protection indicators (i.e., rockfall, avalanche, and landslide protection) due increased timber harvest (Fig. A2.6). In the long term, all protection indicators showed an increasing trend under all harvest intensities. In terms of biodiversity, a synergy occurred due to an increasing tree species diversity. Deadwood amount and number of habitat trees increased under all harvest intensities over time, with the slowest increase occurring for the INC strategy (Fig. A2.7). Further synergies occurred for recreation function (visual attractiveness), where the INC strategy resulted in an improved visual permeation into the stands and an increased tree species diversity (Fig. A2.8), as well as for timber harvest, which increased under the INC strategy (Fig. A2.9).

At the level of overall utility (i.e., the measure for BES multifunctionality), the highest overall utility occurred for the INC strategy (0.631), followed by MED and DEC (0.622 and 0.620 under historic climate, see Fig. A2.11).

## Discussion

### Management effect on disturbance predisposition

Our results showed that the INC strategy caused a reduction of disturbance predisposition for windthrow and bark beetles, which was consistent with findings of other studies about forest management for disturbance mitigation (Zimová et al. 2020; Mathys et al. 2021). However, we found a relatively small effect of increased timber harvest on disturbance predisposition. An important reason for this limited effect was our assumption that the species composition was not actively altered by management and that changes in species composition occurred as an effect of shifts in natural regeneration. As a consequence, the species composition remained largely spruce dominated and hence at a generally high predisposition level in all scenarios. Inducing changes in species composition has been highlighted as an important factor to manage disturbance risk (Jactel et al. 2009). A management approach that actively alters species composition via planting could therefore be a more effective strategy for forest transformation (Mey et al. 2022). However, given the challenging terrain of the case study area, large-scale planting efforts appear unfeasible from an economical as well as ecological point of view and planting efforts will most likely be restricted to recently disturbed stands with high importance for protection (Schwitter et al. 2015). Another reason was that all harvest strategies were assumed to be within the boundaries of a close-to-nature mountain forest management prescribed in Switzerland (Ott et al. 1997; Brang et al. 2014). This type of forest management focuses on relatively small canopy openings (continuous cover forest management) and on emulating natural processes (Bürgi 2015). It thus differs fundamentally from a forest management based on clear-cut systems, which may lead to a faster forest transformation, but deteriorate the protection function (Brang et al. 2006), biodiversity, and other ecosystem services (Larsen et al. 2022). A third aspect to consider was that the occurrence of large-scale disturbances was not simulated in our study, which would cause an additional removal of tall, old, and vulnerable trees. The combined effect of management and disturbance occurrence may thus shift the forest development more effectively towards younger stand ages (Zimová et al. 2020).

Altogether, our simulations showed that the INC strategy has a disturbance mitigation potential (see also Bolte et al. 2009; Jactel et al. 2009; Mathys et al. 2021), but is unlikely to fundamentally revert the entire risk predisposition situation within a high-elevation enterprise currently dominated by tall spruce stands.

### Climate change effects on disturbance predisposition

The most prominent patterns related to changing mean climatic conditions were the different responses of windthrow and bark beetle predisposition, as well as the remarkable increase in bark beetle predisposition when considering a “high-impact” (RCP8.5) climate change scenario.

In regard to bark beetle predisposition, the two “moderate” RC4.5 scenarios (CC22 and CC7) caused only a minor increase in the predisposition indicator, while the impact of the RCP8.5 scenario (CC1) was much stronger. The reason for the small impact of the RCP4.5 scenarios was that the moderate warming did not result in an increase in bark beetle generations and only had a small effect on water supply (i.e., the drought index) for spruce. In contrast, the higher temperatures and decreasing precipitation amounts under the RCP8.5 (CC1) scenario resulted in marked changes for bark beetle generations and water supply in the second half of the twenty-first century, which caused an increase in the predisposition indicator (Temperli et al. 2020). These findings are consistent with the expected behavior of bark beetle outbreaks at high elevations (Bebi et al. 2012) and with results from phenological modeling studies (e.g., Jakoby et al. 2019). Furthermore, they are in line with recent observations showing an intensification of disturbance regimes after the exceptionally hot and dry year 2018 across the EU (Senf and Seidl 2021). An important difference in comparison to other studies is the location of our study enterprise in a high-elevation, temperature-limited environment, where a moderate CC scenario shows less effects on bark beetle dynamics than at lower elevations (Seidl et al. 2008; Zimová et al. 2020). Under a “high-impact” climate change scenario, however, our simulation results underscore that even high-elevation mountain forest enterprises such as Davos could face a much higher risk of increased bark beetle infestations in the future.

In respect to windthrow, the predisposition assessment system accounted for stand conditions, soil, and topography of the stand, which is consistent with other studies about windthrow damages (Hale et al. 2012; Seidl et al. 2014). However, in view of climate change, an increasing tendency of severe storm events was not considered in our study, due to the large uncertainties in the projections of storm events (Seneviratne et al. 2012). This aspect is also important for disturbance interactions since an increase in windthrows with severe storm events leads to more breeding substrate, which amplifies the effect of more bark beetle generations in a warmer climate (Seidl and Rammer 2017). Hence, our projection of climate change effects on windthrow predisposition and its effect on bark beetle disturbances are likely underestimated.

### BES trade-offs and synergies

The management for disturbance risk reduction has traditionally considered wind and bark-beetle interactions; however, trade-offs and synergies with other forest BES have often been ignored (Hlásny et al. 2021a). Considering the increasing demands for BES (Price et al. 2011), a more comprehensive and regionally adapted focus is of key importance for long-term planning (Mina et al. 2017). In our study, the reduction of disturbance predisposition via the INC strategy resulted in trade-offs with carbon sequestration and with the protection function. For carbon sequestration, the main carbon storage occurred within the living biomass (“in situ”), resulting in the slowest build-up under the INC strategy. This trade-off was partly compensated by the increasing amount of carbon stored in wood products (most importantly in the “sawnwood” product class used for long-lasting construction wood, IPCC 2014) and substitution effects (“ex situ”) as a consequence of more timber harvest (Taverna et al. 2007). It has to be emphasized, however, that aiming at an increase of carbon stock in living biomass only is not feasible in the long term since it can rapidly turn into a carbon source once a stand is impacted by a disturbance (Thom and Seidl 2016) and ignores the increasing demand in timber production (Nabuurs et al. 2017; Temperli et al. 2017). In terms of the protection function, the INC strategy slightly decreased the protection against the three gravitational hazards (rockfall protection, avalanches, and landslides). This is reasonable, considering that a reduction of trees of large diameters decreases rockfall protection (Dorren et al. 2004; Bebi et al. 2016). Besides, a reduction in canopy cover has been shown to increase the probability of avalanche initiation and landslides (Sebald et al. 2019). A forward-looking protection forest management therefore needs to pay particular attention to this trade-off and aim at balancing short-term protection efficiency with long-term disturbance mitigation.

Disturbance mitigation via the INC strategy also resulted in synergies with other BES. Besides timber provisioning, where a synergy is evident with increasing harvest intensity, a positive effect occurred for biodiversity indicators, in particular for tree species diversity. Tree species diversity increased the most under a “high-impact” climate change scenario due to an increasing share of broadleaved tree species, which is in accordance with studies about species range expansion under climate change (Vitasse et al. 2012). It has to be emphasized, however, that the biodiversity indicators focused primarily on structural attributes rather than on a species level. When focusing for instance specifically on saproxylic (i.e., deadwood dependent) species, which account for 20–25% of all temperate forest species (Stokland et al. 2012), an increase in harvest intensity which decreases deadwood amount could have substantial negative effects for biodiversity (Gossner et al. 2013; Haeler et al. 2021). Therefore, we recommend an inclusion of indicators for target species, early-successional insects, herbaceous species, and deadwood species (Hilmers et al. 2018) in further studies. For recreation (i.e., visual attractiveness), a synergy with disturbance mitigation was mainly due to a decreasing stand density index, which represents the human preference for stands which are neither too open (Kangas and Niemelainen 1996) nor too dense (Gundersen and Frivold 2008). Furthermore, the increasing tree species diversity improved visual attractiveness, which is supported by a European-wide study of Edwards et al. (2012).

Altogether, our results highlight the enterprise-specific trade-offs and synergies, which can be substantially different from other forest enterprises in mountain regions (Mina et al. 2017; Thrippleton et al. 2021), thereby underlining the importance of accounting for the enterprise-specific environmental conditions, management, and societal preferences (Hlásny et al. 2021a).

### Limitations of the simulation approach

When considering the simulated forest development under climate change and its effect on ecosystem service and disturbance predisposition, a number of limitations and uncertainties should be taken into account.

Regarding the impact of the climate change scenarios, it was notable that the simulated forest dynamics did not show impacts of drought (e.g., increased deadwood amount). Although droughts are currently rare in the case study region, their occurrence is becoming increasingly likely with climate change (Petter et al. 2020). The underestimation of simulated drought effects can be explained by the temporal resolution of the climatic input data (5-year averages) of the forest model SwissStandSim, which is not designed to represent climatic extreme events, but rather general trends under changing mean climatic conditions. Climatic extreme events, such as severe droughts could trigger large-scale forest dieback in the study area, which would lead to more breeding substrates for bark beetles as well as drastic reductions of the protection function against gravitational hazards and other ecosystem services (Elkin et al. 2013; Hlásny et al. 2021b). Besides, the occurrence of climatic extreme events could shift the tree species composition and thereby alter ecosystem functioning in the long term (Anderegg et al. 2013).

In view of the impact of climate change on disturbance predisposition, it is important to note that the occurrence of large-scale disturbances was not represented in the simulations. This has important implications since disturbances reduce the standing stock and typically induce windows of opportunity for tree regeneration (Schumacher et al. 2004). As recently shown by Sommerfeld et al. (2021), these processes lead to conditions which are less conducive for further bark beetle outbreaks in the long term. Using a process-based forest landscape model (e.g., iLand, LandClim) would allow to represent effects of extreme climatic events (Bugmann et al. 2019) and incorporate disturbance dynamics in a self-emergent, dynamic way (Elkin et al. 2013; Seidl and Rammer 2017). However, this model type features a much more complex model structure and requires considerable computational efforts since random effects of disturbance occurrence and disturbance spread have to be accounted for in an iterative, spatially explicit manner (Temperli et al. 2013; Seidl and Rammer 2017). Due to these constraints, a dynamic simulation of disturbances was not feasible within the scope of the DSS, which aims at a reduced level of structural complexity (Vacik and Lexer 2014; Thrippleton et al. 2021).

Besides model choice, other uncertainty sources can play an important role for simulating future forest development (Petter et al. 2020). Previous studies addressing this topic emphasized that differences in climate model chains represent a particular source of uncertainty (Snell et al. 2018). At higher elevations, uncertainties in soil conditions have furthermore been identified as an important uncertainty factor, underlining the need for further research to provide better soil information (Huber et al. 2021).

## Conclusion

Enterprise-level assessments of climate change effects on biodiversity and ecosystem service provisioning and disturbance risk are of key importance for planning forest management. Here, we presented a multi-criteria decision support system, which operates at the scale of a forest enterprise and accounts for BES and disturbance predisposition by windthrow and bark beetles. Its application to a typical high-elevation spruce forest in the Central European Alps demonstrated that increasing harvest intensity via a close-to-nature forestry has the potential to mitigate disturbance predisposition in the long term. However, it also showed that this mitigation effect was by far outweighed by the effect of a “high-impact” climate change scenario. It is hereby important to note that the impacts of large-scale disturbances and extreme climatic events on forest dynamics were not simulated. The results nevertheless underline the crucial importance of reducing greenhouse gas emissions and reaching the goals of the Paris Agreement to avoid detrimental impacts on mountain forests, their biodiversity, and ecosystem service provisioning. In respect to the planning situation for stakeholders, the MCDSS can play an important role to raise awareness of climate change effects on disturbance risk, which may be underestimated in presently little affected enterprises. By integrating a multi-criteria decision analysis, the MCDSS allows stakeholders a combined planning of disturbance mitigation and a comprehensive assessment for trade-offs and synergies with BES, which represents an increasingly important aspect for future forest planning.

## Supplementary Information

Below is the link to the electronic supplementary material.

Supplementary file1 (DOCX 2097 KB)

Supplementary file2 (DOCX 2248 KB)

## Data Availability

The underlying datasets of this study are available from the corresponding author upon request.
