# Supplementary material for: Balancing disturbance risk and ecosystem service provisioning in Swiss mountain forests: an increasing challenge under climate change
Source: Reg Environ Change. 2023 Jan 23;23(1):29. doi: 10.1007/s10113-022-02015-w (PMC9870838; doi:10.1007/s10113-022-02015-w)
Supplement: Supplementary file 1 — Supplementary file1 (DOCX 2097 KB) [file 10113_2022_2015_MOESM1_ESM.docx]

**Appendix – Electronic supplementary materials**

**Article title:** Balancing disturbance risk and ecosystem service provisioning in Swiss mountain forests: an increasing challenge under climate change

**Author names:** Timothy Thrippleton*, Christian Temperli, Frank Krumm, Reinhard Mey, Jürgen Zell, Sophie Stroheker, Martin M. Gossner, Peter Bebi, Esther Thürig, Janine Schweier

***Corresponding author:** Timothy Thrippleton **(**timothy.thrippleton@wsl.ch), Sustainable Forestry, Forest Resources and Management, WSL Birmensdorf, Switzerland

**ESM A1: Extended material and methods**

**A1.0 Forest model SwissStandSim**

SwissStandSim is an empirical, climate-sensitive individual-tree model for the prediction of stand-scale forest development in Switzerland (Zell et al. 2020). It was developed from empirical measurements of 374 stands with 574 000 individual tree records, covering time series of 15 to 112 years. Demographic processes are represented in terms of statistical models for (1) ingrowth (regeneration), (2) growth and (3) mortality at the tree species level. For ingrowth (i.e. regeneration), a new light- and temperature-dependent ingrowth routine was developed by Mey et al. (2022), based on the national forest inventory of Switzerland (Brändli et al. 2020).

In terms of tree species, 11 species or species groups are represented in the model: *Fagus sylvatica*, *Acer* sp., *Quercus* sp., *Picea abies*, *Abies alba*, *Pinus sylvestris*, *Larix decidua*, *Pseudotsuga menziesii*, as well as species groups of ‘long-lived broadleaved’, ‘short-lived broadleaved’ and ‘other conifers’ (Zell 2016). Notably, spruce is the species with the strongest empirical foundation in the model, covering > 190 000 individual tree records. In terms of harvest, SwissStandSim features a range of typical management interventions in Switzerland (see also Mey et al. 2022), for which the user can define beginning, end, intervals and intensity of harvest (defined as relative basal area to be removed per intervention). The model provides individual tree results (e.g. species, diameter at breast height, height, age, volume) for each stand with a resolution of 5 year intervals.

Overall, the model is more detailed in its representation of demographic processes than in other comparable models (e.g., Silva, Pretzsch et al. 2002; BWINPro, Hansen and Nagel 2014). The explained variance in predicting independent data for tree growth is relatively large (74% for beech, 62% for spruce and 68% for fir) when compared to a tree growth model by Rohner et al. (2018) based on the Swiss national forest inventory (43% for beech, 37% for spruce and 53% for fir). With its strong empirical foundation, SwissStandSim is furthermore suitable for conditions of mixed-species forests across a large range of elevations in Switzerland, and allows applications in a climate-change context (Zell et al. 2020).

**A1.1 Stand initialization approach**

Due to the restricted spatial resolution of the forest inventory data in the canton of Grisons (500 m x 500 m), information about stand structure and composition was only available for a section of the stands (i.e., the smallest planning and silvicultural treatment unit). In order to represent all stands of the enterprise, a ‘representative stand type’ (RST) approach was chosen, following the description of Lexer (2013). Based on the stand map provided by the forest enterprise (Stadler et al. 2015), each stand was categorized according to (1) tree species composition (predominant tree species, i.e. *Picea abies* and *Larix decidua*), (2) developmental stage (differentiating between juvenile forest, pole timber and sawtimber) and (3) topography in terms of aspect (south and north-facing slopes) and elevation (upper montane, subalpine, upper subalpine elevation belts). This approach resulted in 36 RSTs, which is a reasonable number for a mountain enterprise (Lexer 2013). For each RST, inventory points from the same RST within the MAB region (Wildi and Ewald 1986) were used to create complete stand datasets, using the statistical approach of Mey et al. (2021), described in detail in Thrippleton et al. (2021).

For the simulations with the forest model, each stand within the enterprise was simulated, using the RST dataset to initialize the stand, as well as local environmental conditions (i.e., slope, aspect and soil conditions at the location of the stand) and climate data (see description below). Notably, stands categorized as ‘bare ground’ and ‘shrub forest’ (e.g., near tree-line) were excluded from the simulation. Furthermore, stands which exceeded the observed volume estimation by more than 25% (based on estimations provided in the stand map, Stadler et al. 2015) were corrected to match the observation by reducing stem numbers (equally over all DBH classes to maintain the same forest structure).

**A1.2 Protection indicators**

In mountain areas with steep slopes (> 35°), gravitational hazards pose a significant threat for settlements and infrastructure. The prime function of many mountain forests is therefore the protection against these hazards (Brang et al. 2006), since trees provide obstacles for falling stones, prevent the release of mass movements and their downslope propagation (Cordonnier et al. 2014). In comparison to technical protection measures, forests provide a cost-effective and ecologically highly valuable solution. Based on natural hazard target profiles for central European protection forests (e.g., Frehner et al. 2005) a set of indicators were developed (Cordonnier et al. 2014), which have been applied in mountain forests across Europe (e.g., Langner et al. 2017; Mina et al. 2017; Pardos et al. 2017).

**A1.2.1 Rockfall protection index (RPI)**

The assessment of the protection effect against rockfalls is based on the concept of the probably residual hazard (PRH), which represents the percentage of rocks that pass through the forested transit zone without being stopped (Cordonnier et al. 2014). PRH is calculated as the ratio of current energy dissipation ability of the stand (CED) and the dissipating maximum energy developed by the falling rock (DME):

$$RPI=1-PRH=1- \frac{CED}{DME}$$

Based on the principles of the tool Rockfor^Net^ (Berger and Dorren 2007), empirical equations to calculate the PRH were derived by Cordonnier et al. (2014).

According to this approach, and in case the basal area of the stand is ≥ 10 m^2^ ha^-1^, the equation is:

$$A= \frac{\left( \theta_{rock}\times N\times250\times\cos\left( slope^{\circ} \right) \right)\times(EvG+\left( DcD \times1.7 \right))\times38.7\times{DBH}^{2.31}}{3.352 \times{10}^{4}\times\left( {0.5\times\rho\times\pi\times(\theta_{rock}/2)}^{3} \times\left( min\left( \sqrt{(2\times9.81\times(F_{ih}+(\frac{250}{\cos\left( slope^{\circ} \right)})\times(tan \left( slope^{\circ} \right)-0.6)))} ; 0.64\times slope^{\circ} \right)^{2} \right)+0.25\times\rho\times\pi\times{(\theta_{rock}/2)}^{3}\times F_{ih} \right)}$$

$$PRH=max(0.01;1-A)$$

If the basal area of the stand is < 10 m^2^ ha^-1^, then the equation is:

$$B= \frac{\left( \theta_{rock}\times N\times250\times\cos\left( slope^{\circ} \right) \right)\times(EvG+\left( DcD \times1.7 \right))\times38.7\times{DBH}^{2.31}}{3.352 \times{10}^{4}\times\left( {0.5\times\rho\times\pi\times(\theta_{rock}/2)}^{3} \times\left( min\left( \sqrt{(2\times9.81\times(F_{ih}+(\frac{250}{\cos\left( slope^{\circ} \right)})\times(tan \left( slope^{\circ} \right)-0.6)))} ; 0.8\times slope^{\circ} \right)^{2} \right)+0.25\times\rho\times\pi\times{(\theta_{rock}/2)}^{3}\times F_{ih} \right)}$$

$$PRH=max(0.01;1-B)$$

With ϴ_rock_ being the diameter of the rock (m), ρ the rock density (kg per m^3^), F_ih_ the initial height of the cliff (m), N the stem density per ha, DBH the average diameter in cm, EvG the percentage of evergreen species (%) and DcD the percentage of deciduous species (%), slope° the slope value in degree. The following assumptions regarding the rock parameters were made based on Wehrli et al. (2006): ϴ_rock_ of 1.24 m, ρ of 2800 kg /m^3^ and F_ih_ of 40 m.

$$RPI=1-PRH$$

**A1.2.2 Avalanche protection index (API)**

The API approach is conceptually similar to the RPI, as it is based on a ratio between current stand parameters and optimal stand parameters required for highest protection. The API uses the basal area of the stand (G) with a given mean diameter (DBH) as a measure to estimate the effect of snow interception on the snow cover stabilization as well as mechanical anchorage effects of the stand to prevent avalanche release (Cordonnier et al. 2014):

For pure evergreen coniferous stands, the API is calculated as:

$$API=min\left( \frac{G}{\left( 0.2901\times DBH+1.494 \right)\times(0.1333\times slope^{\circ}-3)};1 \right)$$

For mixed and pure deciduous stands, the API is calculated as:

$$API=min\left( \frac{G}{\left( 0.528\times DBH+1.5566 \right)\times(0.1333\times slope^{\circ}-3)};1 \right)$$

Notably, avalanches are assumed to occur only at elevations > 800m and at slopes > 28° (Cordonnier et al. 2014).

**A1.2.3 Landslide protection index (LPI)**

The landslide and erosion protection index assumes a direct relationship of protection with forest canopy cover, as trees provide mechanical reinforcement of the soil and prevents erosion events through interception, transpiration and enhancing soil permeability (Cordonnier et al. 2014; Sebald et al. 2019) .Based on the recommendations of Frehner et al. (2005), a forest cover of 30% provides low landslide protection, a value of 30-60% intermediate protection and values > 60% a high protection (considering the projected canopy cover area for all trees with DBH > 5 cm).

**A1.3 Assessment of disturbance predisposition**

In order to assess the disturbance predisposition by windthrow and bark beetles, the predisposition assessment system (PAS) developed Netherer and Nopp-Mayr (2005) and adapted for dynamic modelling by Temperli et al. (2020) was used. The system by Netherer and Nopp-Mayr (2005) was designed to assess disturbance hazard by accounting for the complex relationships between site- and stand-related characteristics, as well as the interaction between windthrow and bark beetle risk. The indicators for stand and site-dependent predisposition were derived from an extensive literature survey and incorporated in the assessment system using expert-based weightings for each predisposition indicator (Fig.A1.3.1). Since its development, the PAS has been applied and evaluated in several studies at different spatial scales in forest stands in Central Europe (Seidl et al. 2007; Temperli et al. 2013; Jakoby et al. 2019; Netherer et al. 2019; Temperli et al. 2020).

For predisposition to storms (PS, Fig.A1.3.1a), the stand-related indicators were proportion of spruce (PS_spruce_) and dominant height (PS_hdom_), i.e. the average height of the 100 largest trees per ha (m), and the site-related indicators were topographic exposure to potential storm corridors (PS_Topex_) (see Quine and White 1998) and slope position (%) (PS_slope_) based on the digital terrain map of Switzerland (© Swisstopo), as well as coarse matter content (> 2 mm) (PS_coarse_) and pH of the soil (PS_pH_), based on the soil suitability map of Switzerland (FSO 2012) and the MAB soil map (Wildi and Ewald 1986).

For predisposition to bark beetles (PB, Fig.A1.3.1b), the stand-related predisposition indicators were proportion of spruce (PB_spruce_), dominant diameter of spruce (PB_ddomspr_) i.e. the average diameter of the 100 largest trees per ha (cm) and stand-related storm predisposition (PS_stand_). For the site-related predisposition, radiation (PB_rad_) (Temperli et al. 2020), slope position (PB_slope_) based on the topographic position index (Wilson et al. 2007; Stadelmann et al. 2014), site-related storm predisposition (PS_site_), as well as soil coarse matter content (> 2 mm) (PB_coarse_) and soil depth (cm) (PB_depth_) based on the soil suitability map of Switzerland (FSO 2012) and the MAB soil map (Wildi and Ewald 1986), a water supply (PB_dr_), based on a site-specific soil water balance (Temperli et al. 2013; Temperli et al. 2020), and the number of beetle generations (PB_gen_) were considered. Due to the direct climatic influence on the indicators PB_dr_ and PB_gen_, both indicators are described in more detail below.

The resulting indicator values were standardized to a range between 0 (low predisposition) and 1 (high predisposition) to allow comparability, using the conversion functions described in Temperli et al. (2020), with exception of the indicator PB_ddomspr_, where the functional relationship of Opiasa (2016) for spruce with intermediate crown length was based used.


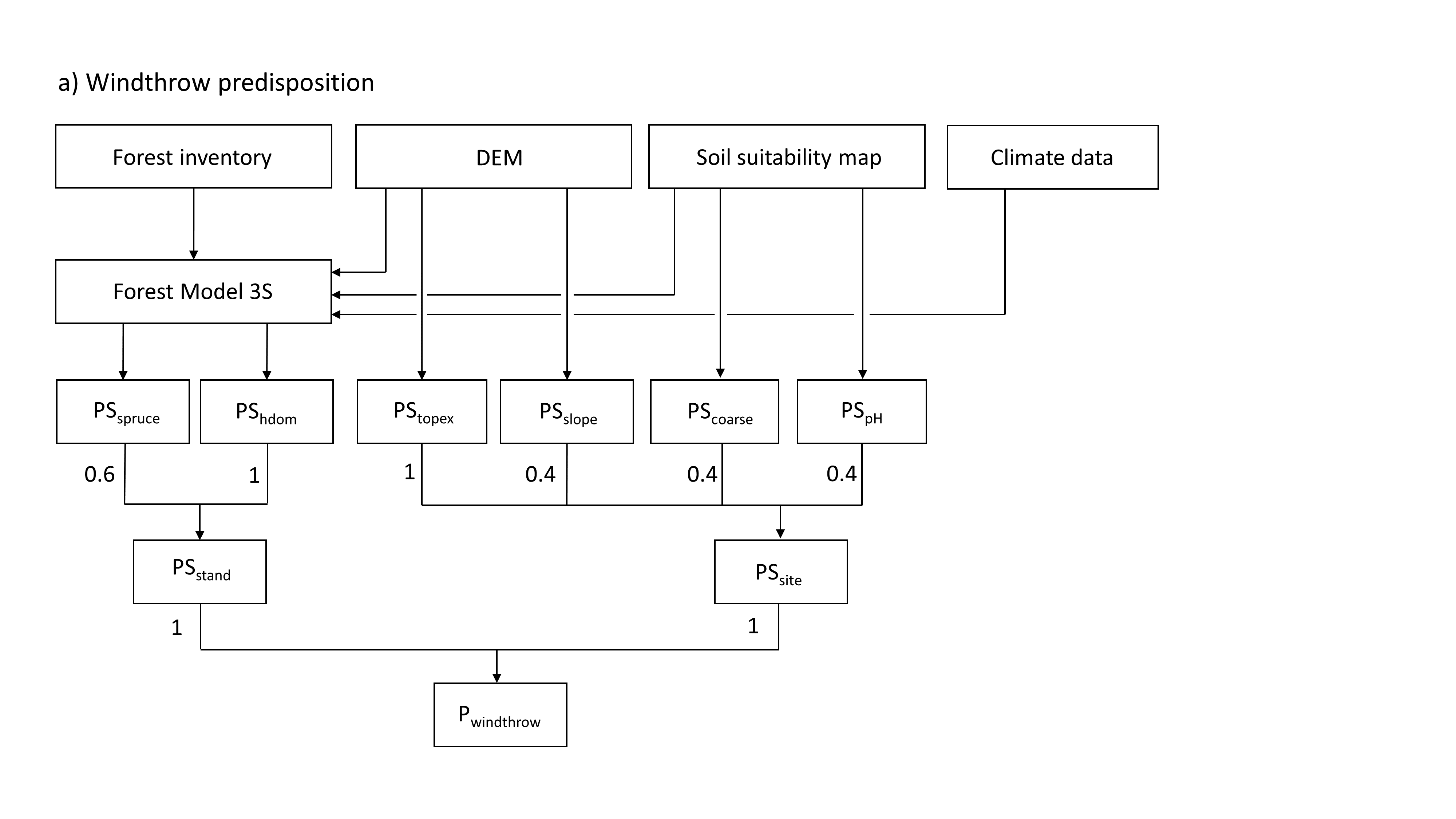

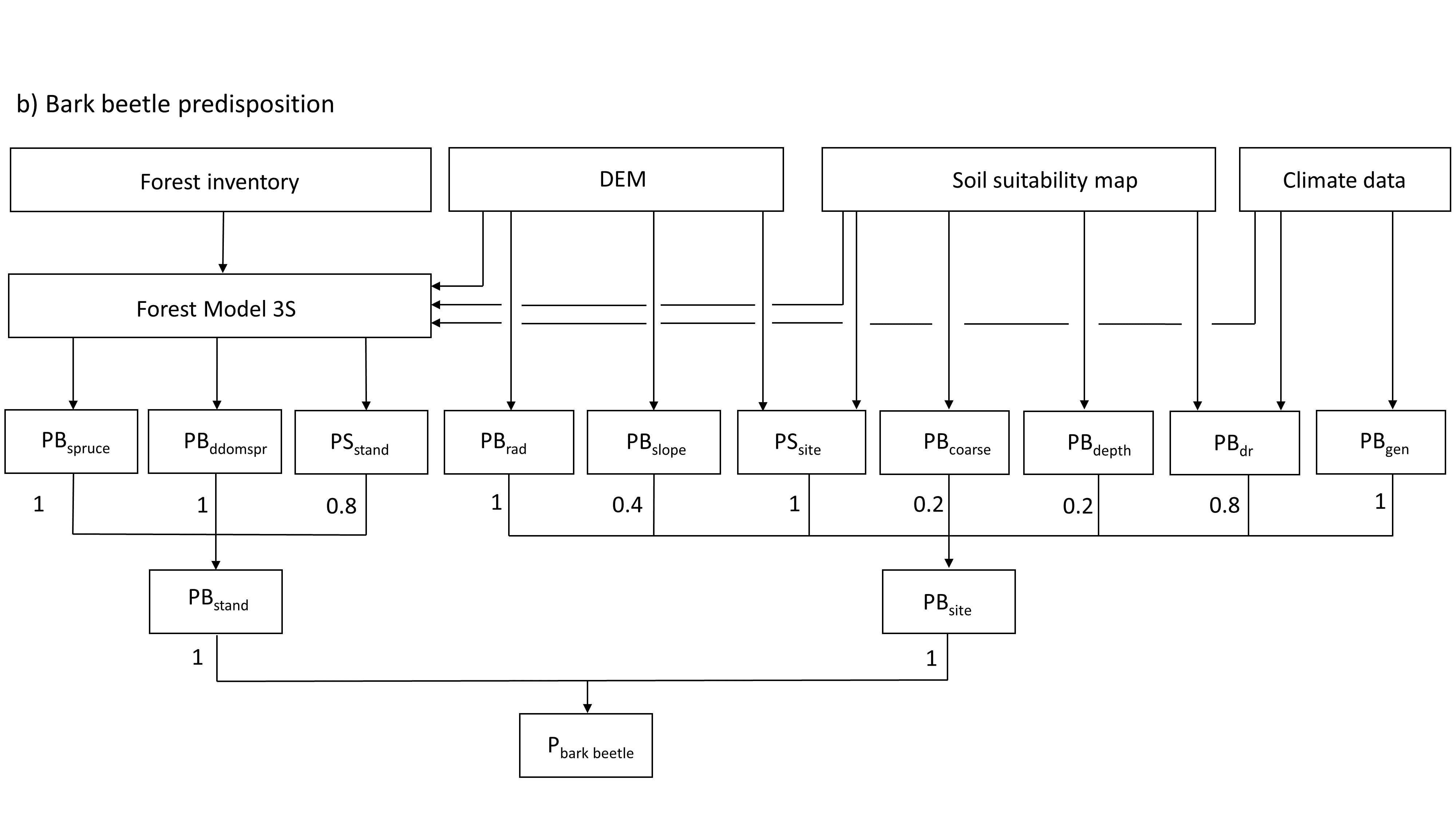


**Fig. A1.3.1 Structure of the predisposition assessment system for (a) windthrow (PS) and (b) bark beetle (PB) predisposition indicators, calculated from forest model (SwissStandSim, abbreviated as ‘3S’) outputs, digital elevation model (DEM), soil suitability maps (SSM) and climate data. Numbers on connector lines represent expert-based weighting factors, see Netherer and Nopp-Mayr (2005) and Temperli et al. (2020) for details**

*Water supply*

For calculating the site-specific annual drought stress experienced by Norway spruce (*Picea abies*), the model by Bugmann and Cramer (1998) was used, as implemented in Temperli et al. (2020). This approach has been applied in several previous studies to estimate the drought-related predisposition of spruce to bark beetle attacks (Seidl et al. 2007; Temperli et al. 2013; Jakoby et al. 2019). The water balance model calculates a potential evapotranspiration (PET), based on the approach of Thornthwaite and Mather (1957), accounting for temperature, slope and aspect. Actual evapotranspiration (AET) is calculated as a function of PET, precipitation, soil water holding capacity (‘bucket size’) and assumptions about vegetation influence (see Bugmann and Cramer 1998). A drought index is then calculated based on the relationship of AET to PET (see also Federer 1982) and tree species-specific drought tolerance thresholds (Bugmann and Cramer 1998; Temperli et al. 2013). Local soil water holding capacity was estimated based on soil maps of the MAB project (Wildi and Ewald 1986), assuming that a low, intermediate and high water holding capacity corresponds to ‘bucket size’ values of 9, 13 and 17 cm, based on Henne et al. (2011) and Thrippleton et al. (2018).

*Number of bark beetle generations*

To extend the applicability of the disturbance predisposition assessment system towards future climatic conditions, the index ‘number of bark beetle generations’ (PB_gen_) was implemented as a statistical model representing the relationship between the annual degree day sum (AnDD) and the number of bark beetle generations (nGen), based on the results of the phenological model for Switzerland by Jakoby et al. (2016) and Jakoby et al. (2019). Swiss-wide model results for the number of bark beetle generations ranging from historic (1980) to a future ‘high-impact’ climate change scenario (+4.3°C until 2100, scenario CLM) were related to the annual degree day sum, assuming a minimum developmental temperature threshold for *Ips typographus* of 5.12°C and a photoperiod from April to September (Jakoby et al. 2019). The resulting model (Eq. A1.3.1, adj.R^2^ = 0.94) was capable of reproducing main developmental trends of bark beetle generations under climate change (Fig. A1.3.2).

$nGen=-0.837+0.00179\times AnDD-6.288 \times{10}^{-8}\times{ANDD}^{2}$ (Eq. A1.3.1)


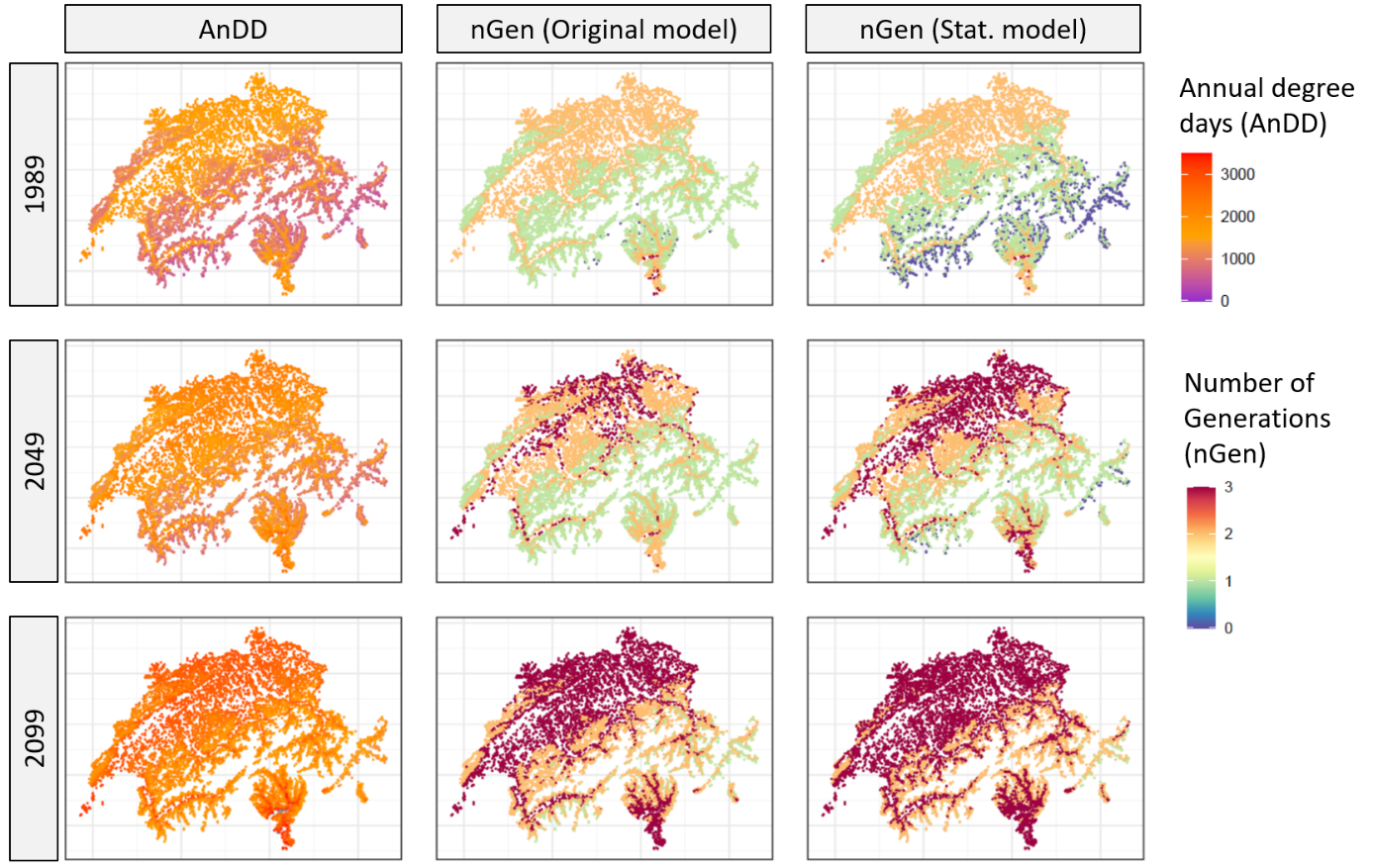


**Fig. A1.3.2 Annual degree day sum (AnDD) and number of bark beetle generations per year (nGen) for original model results by Jakoby et al. (2016) and prediction from statistical model (Eq.A1.3.1) for years 1989, 2049 and 2099 under a ‘high-impact’ (CLM) climate change scenario across Switzerland**

**A1.4 Management strategies**

For studying the effect of different harvest intensities, three strategies (DEC, MED and INC) with different harvest intensity per intervention were defined.

The ‘medium intensity’ (MED) strategy was based on current management intensity, defined as a removal of 35% of basal area at intervals of 30 years via small-scale selective harvest within a close-to-nature forestry framework, which is typical for mountain forests in this region (Bircher et al. 2015), using the ‘Tfa’ harvest type within SwissStandSim (Zell et al. 2020). The forests of the case study area are distinguished into three different priority classes for management: a ‘high-priority’, a ‘medium-priority’ and a ‘low-priority’ area (Fig.A1.4). While the ‘high-‘ and ‘medium-priority’ areas comprise accessible stands and important protection forests, the ‘low-priority’ area contains stands which are managed at a lower intensity due to lower protection importance or limited accessibility (Stadler et al. 2015). It was therefore assumed that ‘high priority’ stands are scheduled first for management interventions (starting in year 2015, i.e. the first simulation timestep), followed by ‘medium priority’ stands (interventions starting in year 2025) and ‘low priority’ (starting in year 2035). Furthermore, a reduced harvest intensity was assumed for the ‘low priority’ stands due to limited accessibility (-15% intensity compared to ‘high’ and ‘medium’ priority).

The DEC and INC strategy assumed the same management setup as in the MED strategy, with exception of management intensity, which was defined as 25% (DEC) and 45% (INC) basal area removal per interval.


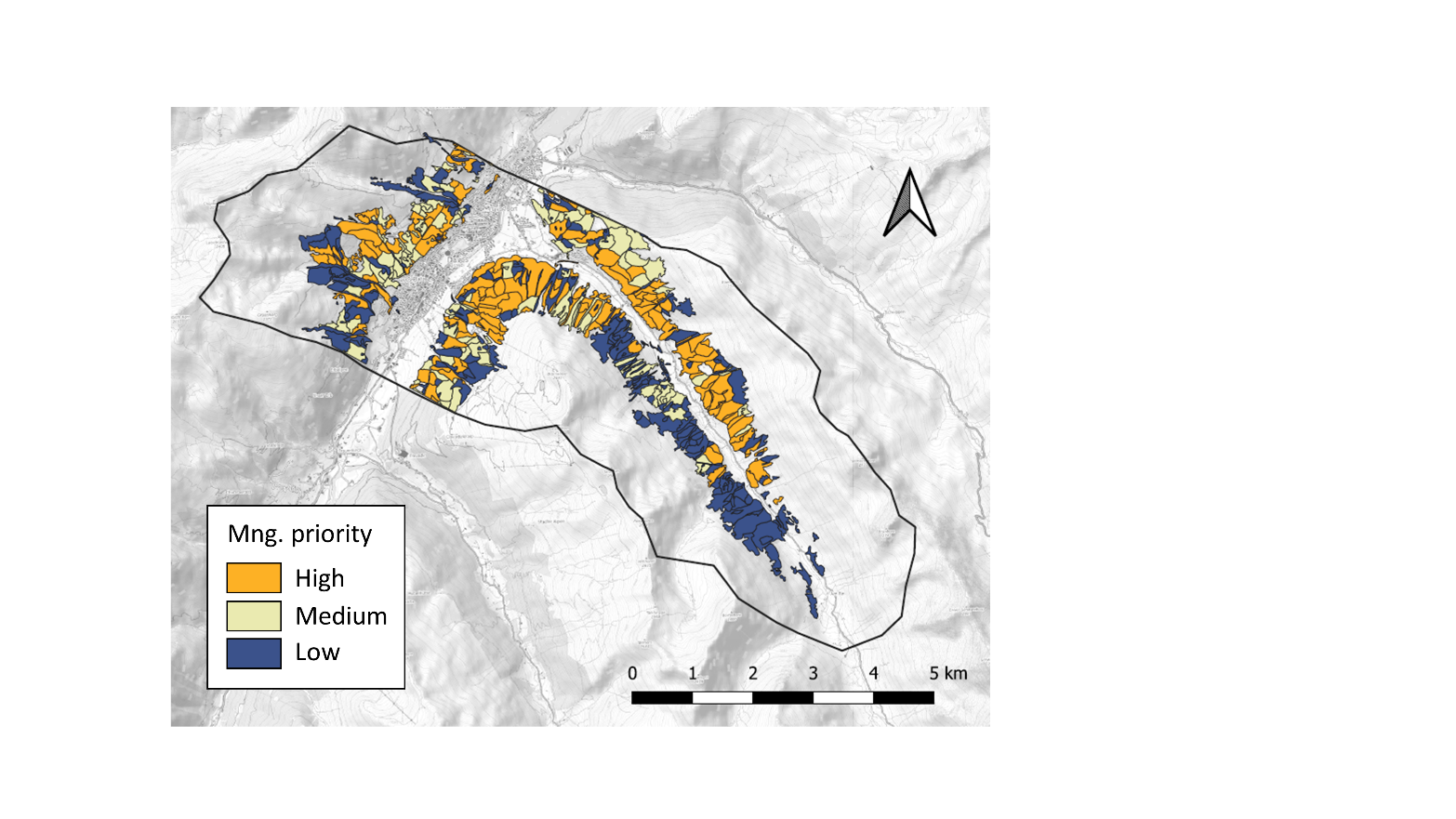


**Fig. A1.4 Stand map showing the management priority classes for case study area Davos**

**A1.5 Climate change scenarios**

For the simulation of present and future climate scenarios, downscaled climate datasets by Brunner et al. (2019) were used for the location of Davos and aggregated to 5 year averages (annual mean temperature, precipitation sum and moisture index), as described in Zell (2016). The climate scenario data by Brunner et al. (2019) were based on representative concentration pathways (RCP) and downscaled using a quantile mapping regional down-scaling approach (Gudmundsson et al. 2012). Following the approach in Thrippleton et al. (2021), historic climate data was derived from the CC22 dataset of Brunner et al. (2019) for the reference period 1981 to 2010 and expanded to a climate time series until year 2100 by randomly resampling the climate data. For the climate change scenarios, three scenarios of Brunner et al. (2019) representing typical ‘dry’ (CC1), ‘medium’ (CC22) and ‘wet’ (CC7) future climate were used for the time 2010 to 2100. An overview over the climate change scenarios is provided in Table A1.5.1, for technical details about the datasets, cf. Brunner et al. (2019). In order to account for changing climatic conditions with elevation, a lapse-rate approach was used to calculate climatic conditions at each stand, assuming the temperature and precipitation lapse rates of Schumacher et al. (2004) for Dischma valley.

**Table A1.5.1 Selected climate change (CC) scenarios from Brunner et al. (2019) for the case study area Davos. Values for ΔT and ΔAP indicate the change in mean annual temperature (in °C) and annual precipitation sum (in %) from 2070 to 2100 relative to mean values from the historic reference period (1981–2010). Abbreviations are: GCM: global climate model, RCM: regional climate model, RCP: representative concentration pathway (see CH2018(2018).**

| Climate Scenario | Wet | Medium | Dry |
| --- | --- | --- | --- |
| Code (Brunner et al. 2019) | CC7 | CC22 | CC1 |
| GCM | ICHEC-EC-EARTH | ICHEC-EC-EARTH | MOHC-HadGEM2 |
| RCM | DMI-HIRHAM5 | SMHI-RCA4 | CLMcom-CCLM4 |
| Resolution | EUR11 | EUR44 | EUR44 |
| RCP | 4.5 | 4.5 | 8.5 |
| Δ T (°C) | 2.46 | 1.51 | 4.37 |
| Δ AP (%) | +11.6 | +8.4 | -8.91 |

**A1.6 Weighting factors for indicators**

**Table A1.6 Weighting factors for biodiversity and ecosystem services (BES) as well as disturbance mitigation, assigned at the level of indicator groups (λ_a_) and individual indicators (λ_a,i_). Estimations are based on Cathomen and Vanoni (2020) for indicator groups as well as AWN (2018) and Stadler et al. (2015) for individual indicators**

| BES group | λ_a_ |  | BES indicators | Reference | λ_a,i_ |
| --- | --- | --- | --- | --- | --- |
|  |  |  |  |  |  |
| Carbon sequestration | 0.06 |  | Carbon sequestration | Blattert et al. (2018), Thrippleton et al. (2021) | 1.00 |
|  |  |  |  |  |  |
| Biodiversity | 0.17 |  | Shannon index alpha | Shannon and Weaver (1949) | 0.17 |
|  |  |  | Shannon index gamma | Shannon and Weaver (1949) | 0.17 |
|  |  |  | PostHoc index alpha | Staudhammer and LeMay (2001) | 0.13 |
|  |  |  | PostHoc index gamma | Staudhammer and LeMay (2001) | 0.13 |
|  |  |  | Amount of deadwood | Blattert et al. (2018), Thrippleton et al. (2021) | 0.22 |
|  |  |  | Number of habitat trees | Blattert et al. (2018), Thrippleton et al. (2021) | 0.17 |
|  |  |  |  |  |  |
| Recreation | 0.17 |  | Height of largest trees | Edwards et al. (2012) | 0.20 |
| (visual attractiveness) |  |  | Variation in tree height | Edwards et al. (2012) | 0.10 |
|  |  |  | Diversity of tree species | Edwards et al. (2012) | 0.15 |
|  |  |  | Canopy cover | Edwards et al. (2012) | 0.15 |
|  |  |  | Visual permetation | Edwards et al. (2012) | 0.20 |
|  |  |  | Deadwood (harvest residue) | Edwards et al. (2012) | 0.10 |
|  |  |  | Deadwood (natural mortality) | Edwards et al. (2012) | 0.10 |
|  |  |  |  |  |  |
| Timber provisioning | 0.17 |  | Timber harvested | Thrippleton et al. (2021) | 0.36 |
|  |  |  | Productivity | Thrippleton et al. (2021) | 0.27 |
|  |  |  | Sustainability timber prod. | Thrippleton et al. (2021) | 0.18 |
|  |  |  | Growing stock | Thrippleton et al. (2021) | 0.18 |
|  |  |  |  |  |  |
| Protection | 0.28 |  | Rockfall protection index | Cordonnier et al. (2014), Thrippleton et al. (2021) | 0.38 |
|  |  |  | Avalanche protection index | Cordonnier et al. (2014), Thrippleton et al. (2021) | 0.38 |
|  |  |  | Landslide protection index | Cordonnier et al. (2014), Thrippleton et al. (2021) | 0.23 |
|  |  |  |  |  |  |
| Disturbance mitigation | 0.17 |  | Windthrow | Temperli et al. (2020) | 0.50 |
|  |  |  | Bark beetle | Temperli et al. (2020) | 0.50 |

**References**

AWN (2018) Waldentwicklungsplan 2018+, Herrschaft/Prättigau/Davos (Cantonal forest management plan for Herrschaft/Prättigau/Davos, 2018). Chur, Switzerland.

Berger F, Dorren LKA (2007) Principles of the tool Rockfor net for quantifying the rockfall hazard below a protection forest. 158(6):157–165.

Bircher N, Cailleret M, Huber M, Bugmann H (2015) Empfindlichkeit typischer Schweizer Waldbestände auf den Klimawandel. Schweizerische Zeitschrift für Forstwesen 166(6):408–419.

Blattert C, Lemm R, Thees O, Hansen J, Lexer MJ et al. (2018) Segregated versus integrated biodiversity conservation: Value-based ecosystem service assessment under varying forest management strategies in a Swiss case study. Ecol Indic 95:751-764. doi:10.1016/j.ecolind.2018.08.016.

Brändli U-B, Abegg M, Allgaier Leuch B (2020) Schweizerisches Landesforstinventar. Ergebnisse der vierten Erhebung 2009–2017. Birmensdorf, Eidgenössische Forschungsanstalt für Wald, Schnee und Landschaft WSL. Bern, Bundesamt für Umwelt. doi: 10.16904/envidat.146

Brang P, Schönenberger W, Frehner M, Schwitter R, Thormann J-J et al. (2006) Management of protection forests in the European Alps: an overview. For Snow Landsc Res 80(1):23-44.

Brunner MI, Gurung AB, Zappa M, Zekollari H, Farinotti D et al. (2019) Present and future water scarcity in Switzerland: Potential for alleviation through reservoirs and lakes. Sci Total Environ 666:1033-1047. doi:10.1016/j.scitotenv.2019.02.169.

Bugmann H, Cramer W (1998) Improving the behaviour of forest gap models along drought gradients. Forest Ecology and Management 103(2-3):247-263. doi:10.1016/S0378-1127(97)00217-X.

Cathomen M, Vanoni M (2020) Tamins - Forest management and the woods of a municipality in Switzerland. In: Krumm F, Schuck A, Rigling A, editors. How to balance forestry and biodiversity conservation - A view across Europe. Birmensdorf, Switzerland: European Forest Institute (EFI); Swiss Federal Institute for Forest, Snow and Landscape (WSL); p. 479-488.

CH2018 (2018) CH2018 – Climate Scenarios for Switzerland, Technical Report. National Centre for Climate Services. Zurich, Switzerland.

Cordonnier T, Berger F, Elkin C, Lämås T, M. M (2014) ARANGE Deliverable D2.2 - Models and linker functions (indicators) for ecosystem services (updated version 28.03.2014).

Edwards D, Jay M, Jensen FS, Lucas B, Marzano M et al. (2012) Public preferences for structural attributes of forests: Towards a pan-European perspective. Forest Policy Econ 19:12-19. doi:10.1016/j.forpol.2011.07.006.

Federer CA (1982) Transpirational supply and demand: Plant, soil, and atmospheric effects evaluated by simulation. Water Resources Research 18(2):355-362. doi:10.1029/WR018i002p00355.

Frehner M, Wasser B, Schwitter R (2005). Nachhaltigkeit und Erfolgskontrolle im Schutzwald. Wegleitung für Pflegemassnahmen in Wäldern mit Schutzfunktion, Vollzug Umwelt. Bern, Switzerland: Bundesamt für Umwelt.

FSO 2012. Bodeneignungskarte der Schweiz: Federal Statistical Office. GEOSTAT: Neuchâtel, Switzerland.

Gudmundsson L, Bremnes JB, Haugen JE, Engen-Skaugen T (2012) Technical Note: Downscaling RCM precipitation to the station scale using statistical transformations - a comparison of methods. Hydrol Earth Syst Sc 16(9):3383-3390. doi:10.5194/hess-16-3383-2012.

Hansen J, Nagel J (2014) Waldwachstumskundliche Softwaresysteme auf Basis von TreeGrOSS – Anwendung und theoretische Grundlagen. Beiträge aus der Nordwestdeutschen Forstlichen Versuchsanstalt Vol 11. Universtitätsverlag Göttingen. Göttingen, Germany.

Henne PD, Elkin CM, Reineking B, Bugmann H (2011) Did soil development limit spruce (Picea abies) expansion in the Central Alps during the Holocene? Testing a palaeobotanical hypothesis with a dynamic landscape model. Journal of Biogeography 38:933-949. doi:10.1111/j.1365-2699.2010.02460.x.

Jakoby O, Lischke H, Wermelinger B (2019) Climate change alters elevational phenology patterns of the European spruce bark beetle (Ips typographus). Global Change Biology 25(12):4048-4063. doi:10.1111/gcb.14766.

Jakoby O, Stadelmann G, Lischke H, Wermelinger B (2016) Borkenkäfer und Befallsdisposition der Fichte im Klimawandel. In: Pluess AR, Augustin S, Brang P, editors. Wald im Klimawandel Grundlagen für Adaptationsstategien. Bern; Stuttgart: Bundesamt für Umwelt BAFU; Eidg. Forschungsanstalt WSL; Haupt; p. 447.

Langner A, Irauschek F, Perez S, Pardos M, Zlatanov T et al. (2017) Value-based ecosystem service trade-offs in multi-objective management in European mountain forests. Ecosyst Serv 26:245-257. doi:10.1016/j.ecoser.2017.03.001.

Lexer MJ (2013) ARANGE Deliverable D1.2 - Catalogue of harmonized environmental variables.

Mey R, Stadelmann G, Thürig E, Bugmann H, Zell J (2021) From small forest samples to generalised uni-and bimodal stand descriptions. Methods in Ecology and Evolution 12:634–645. doi:10.1111/2041-210X.13566.

Mey R, Zell J, Thürig E, Stadelmann G, Bugmann H et al. (2022) Tree species admixture increases ecosystem service provisioning in simulated spruce- and beech-dominated stands. Eur J For Res 141:801–820. doi:10.1007/s10342-022-01474-4.

Mina M, Bugmann H, Cordonnier T, Irauschek F, Klopcic M et al. (2017) Future ecosystem services from European mountain forests under climate change. Journal of Applied Ecology 54(2):389-401. doi:10.1111/1365-2664.12772.

Netherer S, Nopp-Mayr U (2005) Predisposition assessment systems (PAS) as supportive tools in forest management - rating of site and stand-related hazards of bark beetle infestation in the High Tatra Mountains as an example for system application and verification. Forest Ecology and Management 207(1-2):99-107. doi:10.1016/j.foreco.2004.10.020.

Netherer S, Panassiti B, Pennerstorfer J, Matthews B (2019) Acute Drought Is an Important Driver of Bark Beetle Infestation in Austrian Norway Spruce Stands. Frontiers in Forests and Global Change 2. doi:10.3389/ffgc.2019.00039.

Opiasa M. 2016. Predisposition of Norway spruce to European spruce bark beetle infestation and spatial development of an outbreak after windthrow. Master Thesis. Zurich, Switzerland: ETH Zurich.

Pardos M, Perez S, Calama R, Alonso R, Lexer MJ (2017) Ecosystem service provision, management systems and climate change in Valsain forest, central Spain. Regional Environmental Change 17(1):17-32. doi:10.1007/s10113-016-0985-4.

Pretzsch H, Biber P, Dursky J (2002) The single tree-based stand simulator SILVA: construction, application and evaluation. Forest Ecology and Management 162(1):3-21. doi:10.1016/S0378-1127(02)00047-6.

Quine CP, White IMS (1998) The potential of distance-limited topex in the prediction of site windiness. Forestry 71(4):325-332. doi:DOI 10.1093/forestry/71.4.325.

Rohner B, Waldner P, Lischke H, Ferretti M, Thürig E (2018) Predicting individual-tree growth of central European tree species as a function of site, stand, management, nutrient, and climate effects. Eur J For Res 137(1):29-44. doi:10.1007/s10342-017-1087-7.

Schumacher S, Bugmann H, Mladenoff D (2004) Improving the formulation of tree growth and succession in a spatially explicit landscape model. Ecological Modelling 180(1):175-194. doi:10.1016/j.ecolmodel.2003.12.055.

Sebald J, Senf C, Heiser M, Scheidl C, Pflugmacher D et al. (2019) The effects of forest cover and disturbance on torrential hazards: large-scale evidence from the Eastern Alps. Environ Res Lett 14(11):1-12. doi:10.1088/1748-9326/ab4937.

Seidl R, Baier P, Rammer W, Schopf A, Lexer MJ (2007) Modelling tree mortality by bark beetle infestation in Norway spruce forests. Ecological Modelling 206(3-4):383-399. doi:10.1016/j.ecolmodel.2007.04.002.

Shannon CE, Weaver W (1949). The Mathematical Theory of Communication. Urbana, Illinois: University of Illinois Press.

Stadelmann G, Bugmann H, Wermelinger B, Bigler C (2014) Spatial interactions between storm damage and subsequent infestations by the European spruce bark beetle. Forest Ecology and Management 318:167-174. doi:10.1016/j.foreco.2014.01.022.

Stadler M, Hefti H, Kessler A, Hubert M, Mattli J (2015) Betriebsplan Gemeindewald Davos. Enterprise management plan of the communal forest of Davos.

Staudhammer CL, LeMay VM (2001) Introduction and evaluation of possible indices of stand structural diversity. Canadian Journal of Forest Research 31(7):1105-1115. doi:10.1139/x01-033.

Temperli C, Blattert C, Stadelmann G, Brändli UB, Thürig E (2020) Trade-offs between ecosystem service provision and the predisposition to disturbances: a NFI-based scenario analysis. For Ecosyst 7(1):1-17. doi:10.1186/s40663-020-00236-1.

Temperli C, Bugmann H, Elkin C (2013) Cross-scale interactions among bark beetles, climate change, and wind disturbances: a landscape modeling approach. Ecological Monographs 83(3):383-402. doi:10.1890/12-1503.1.

Thornthwaite CW, Mather JR (1957) Instructions and tables for computing potential evapotranspiration and the water balance. Publications in climatology 10(3):183-311.

Thrippleton T, Blattert C, Bont LG, Mey R, Zell J et al. (2021) A multi-criteria decision support system for strategic planning at the Swiss forest enterprise level: coping with climate change and shifting demands in ecosystem service provisioning. Frontiers in Forests and Global Change 4:1-18. doi:10.3389/ffgc.2021.693020.

Thrippleton T, Bugmann H, Folini M, Snell RS (2018) Overstorey-Understorey Interactions Intensify After Drought-Induced Forest Die-Off: Long-Term Effects for Forest Structure and Composition. Ecosystems 21(4):723-739. doi:10.1007/s10021-017-0181-5.

Wehrli A, Dorren LKA, Berger F, Zingg A, Schönenberger W et al. (2006) Modelling long-term effects of forest dynamics on the protective effect against rockfall. For Snow Landsc Res 80(1):57–76.

Wildi O, Ewald K (1986) Der Naturraum und dessen Nutzung im alpinen Tourismusgebiet von Davos. Ergebnisse des MAB-Projektes Davos. Berichte Eidgenössische Anstalt für das forstliche Versuchswesen (Vol. 289). Birmensdorf, Switzerland.

Wilson MFJ, O'Connell B, Brown C, Guinan JC, Grehan AJ (2007) Multiscale terrain analysis of multibeam bathymetry data for habitat mapping on the continental slope. Mar Geod 30(1-2):3-35. doi:10.1080/01490410701295962.

Zell J (2016) SwissStandSim: A climate sensitive single tree stand simulator for Switzerland. Swiss Federal Institute of Forest, Snow and Landscape Research WSL. Birmensdorf, Switzerland

Zell J, Nitzsche J, Stadelmann G, Thürig E (2020) SwissStandSim: ein klimasensitives, einzelbaumbasiertes Waldwachstumsmodell. Schweizerische Zeitschrift für Forstwesen 171(3):116 –123.
