# Supplementary material for: Balancing disturbance risk and ecosystem service provisioning in Swiss mountain forests: an increasing challenge under climate change
Source: Reg Environ Change. 2023 Jan 23;23(1):29. doi: 10.1007/s10113-022-02015-w (PMC9870838; doi:10.1007/s10113-022-02015-w)
Supplement: Supplementary file 2 — Supplementary file2 (DOCX 2248 KB) [file 10113_2022_2015_MOESM2_ESM.docx]

**Appendix – Electronic supplementary materials**

**Article title:** Balancing disturbance risk and ecosystem service provisioning in Swiss mountain forests: an increasing challenge under climate change

**Author names:** Timothy Thrippleton*, Christian Temperli, Frank Krumm, Reinhard Mey, Jürgen Zell, Sophie Stroheker, Martin M. Gossner, Peter Bebi, Esther Thürig, Janine Schweier

***Corresponding author:** Timothy Thrippleton **(**timothy.thrippleton@wsl.ch), Sustainable Forestry, Forest Resources and Management, WSL Birmensdorf, Switzerland

**ESM A2: Additional results**


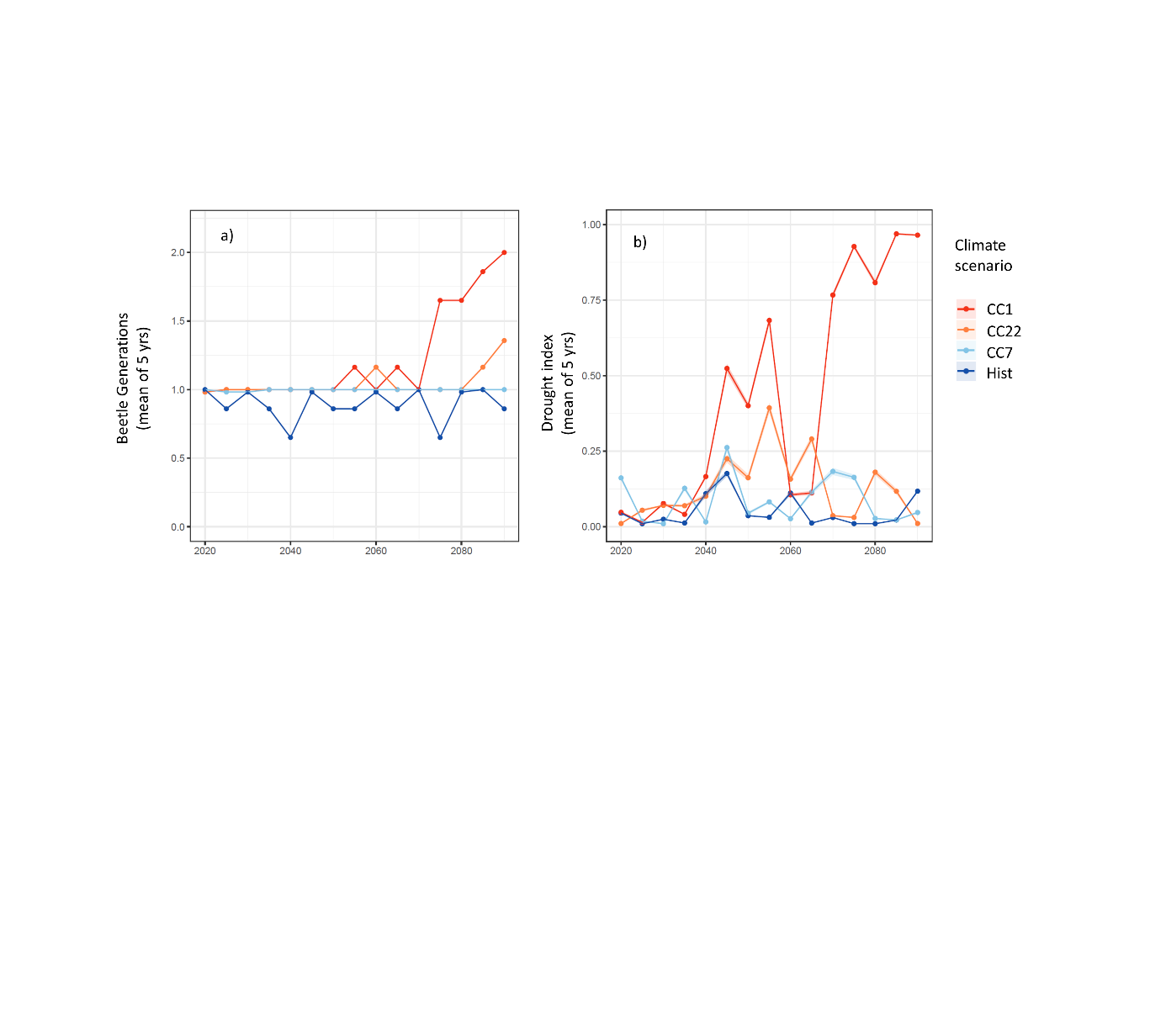


**Fig. A2.1 Effect of climate scenarios (Hist: Historic, CC7: RCP4.5-wet, CC22: RCP4.5-medium, CC1:RCP8.5-dry) on (a) number of bark beetle generations, (b) spruce drought index (mean across all stands of forest enterprise)**


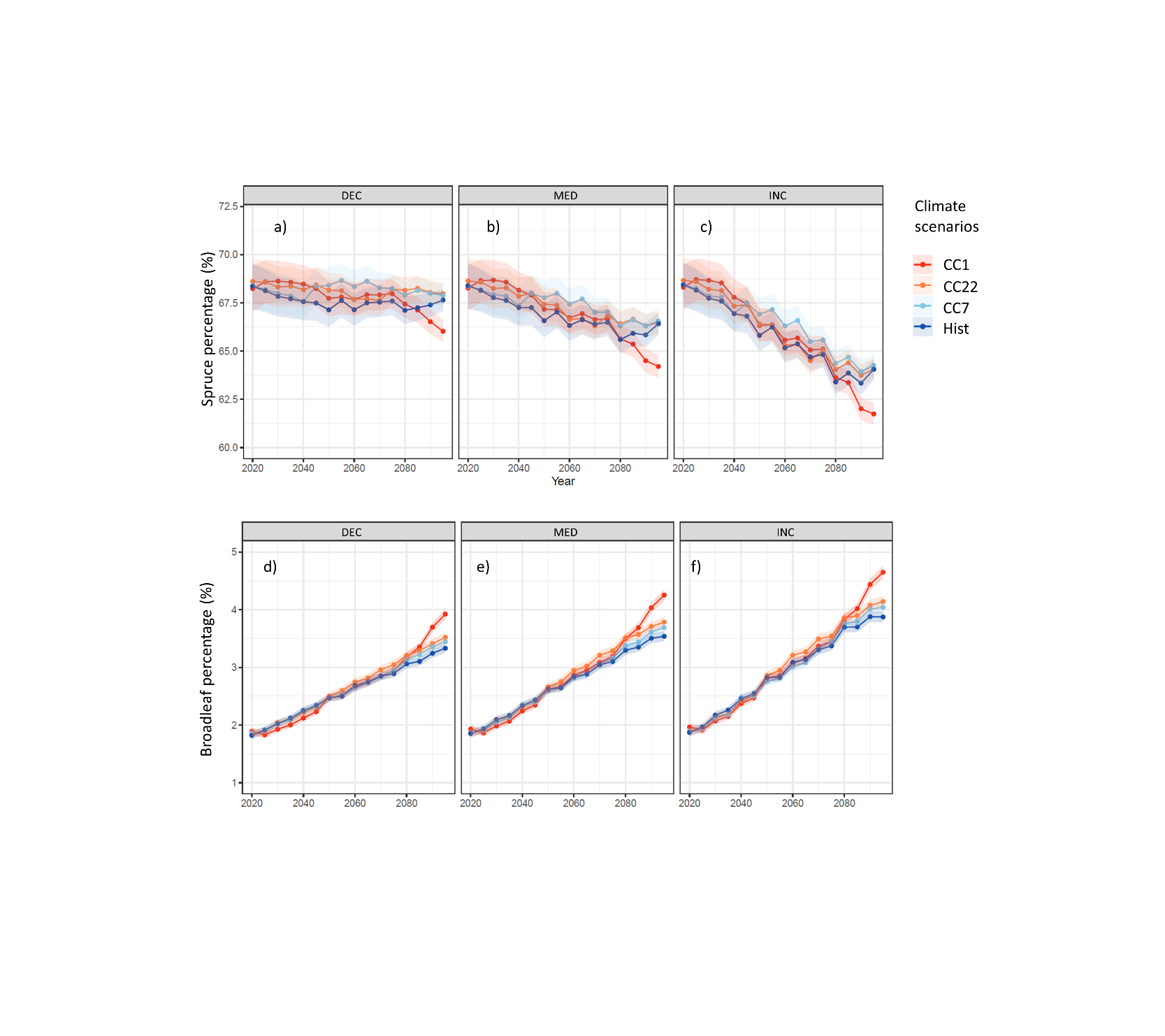


**Fig A2.2 Effect of climate scenarios (Hist: Historic, CC7: RCP4.5-wet, CC22: RCP4.5-medium, CC1:RCP8.5-dry) and management scenarios (DEC, MED, INC) on (a-c) spruce percentage and (d-f) percentage of broadleaved tree species (mean and standard error of the mean across all stands of forest enterprise, indicated by shaded area)**

*
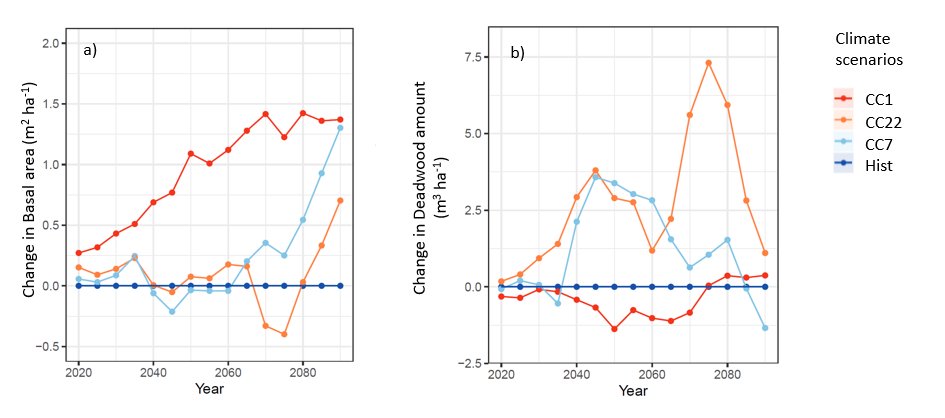
*

**Fig. A2.3 Climate change effect on change in basal area and deadwood amount (mean over all stands in enterprise) for the historic climate (‘Hist’), as well as climate change scenarios (CC7: RCP4.5-wet, CC22: RCP4.5-medium, CC1:RCP8.5-dry) under current management intensity (‘MED’)**

*
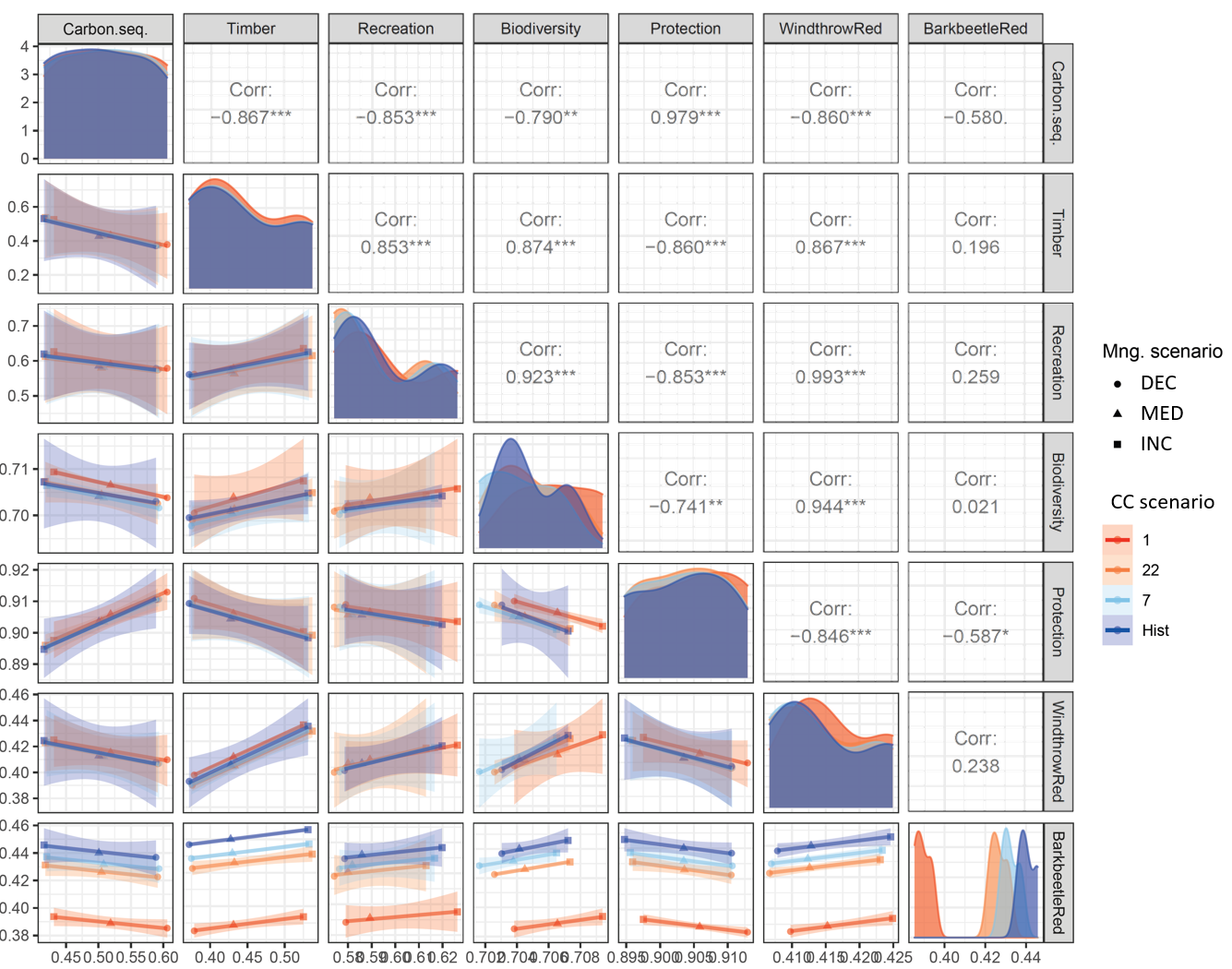
*

**Fig. A2.4 Correlogram with Spearman’s correlation coefficient for all biodiversity and ecosystem service (BES) as well as disturbance mitigation indicators (average across case study enterprise). Points indicate the mean of partial utility over the simulation timespan for the respective management scenario and shaded area indicates the standard error of the model fit. Asterisks indicate p values ≤ 0.05 (*), ≤ 0.01 (**) and ≤ 0.001 (***).**


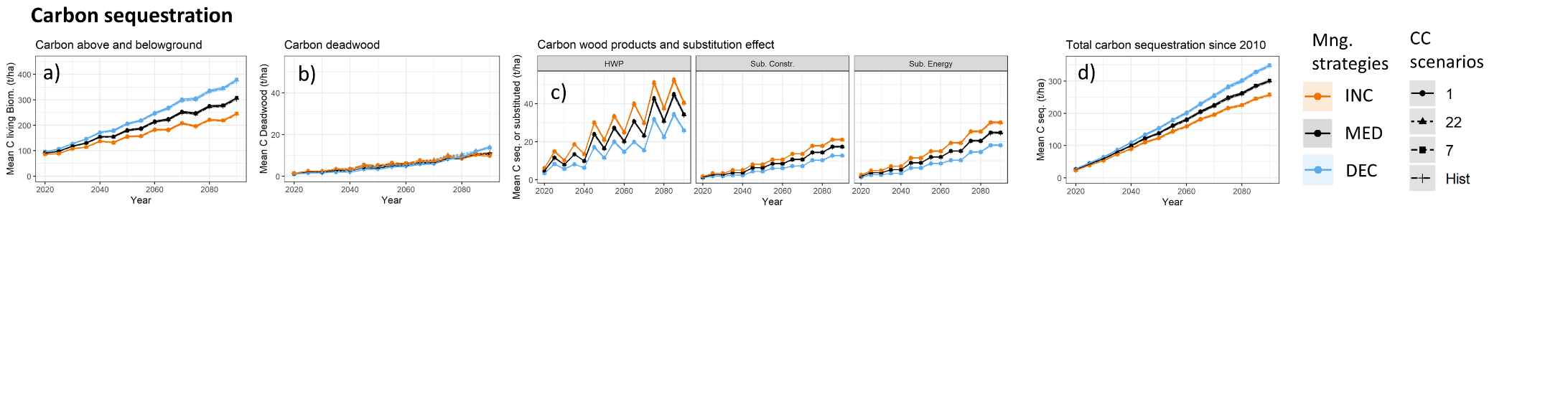


**Fig. A2.5 Indicators for carbon sequestration under different management strategies (DEC: decreased, MED: medium, INC: increased intensity) and climate scenarios (Hist: Historic, CC7: RCP4.5-wet, CC22: RCP4.5-medium, CC1:RCP8.5-dry). Plots show mean and standard error of the mean (indicated by shaded area ) across all stands of forest enterprise.**


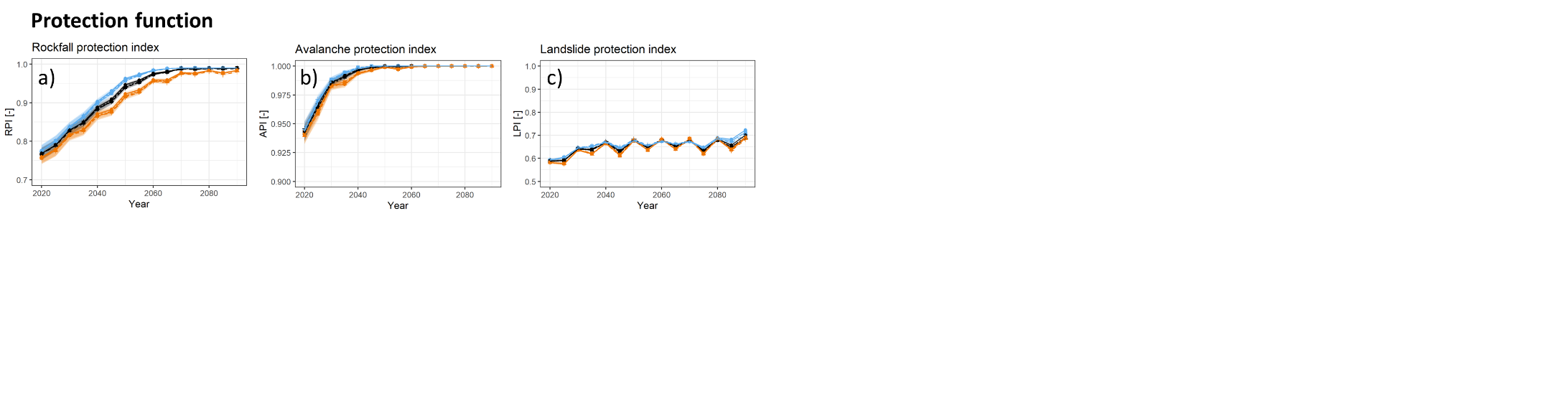


**Fig. A2.6 Indicators for protection function under different management strategies (for abbreviations, see Fig. A2.2)**


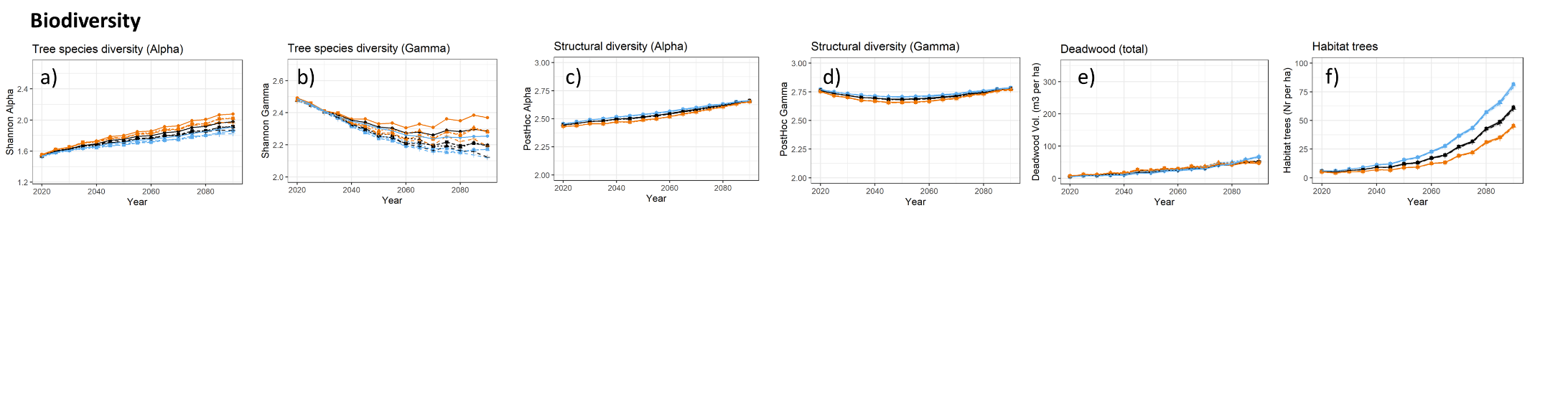


**Fig. A2.7 Indicators for biodiversity under different management strategies and climate scenarios (for abbreviations, see Fig. A2.2)**


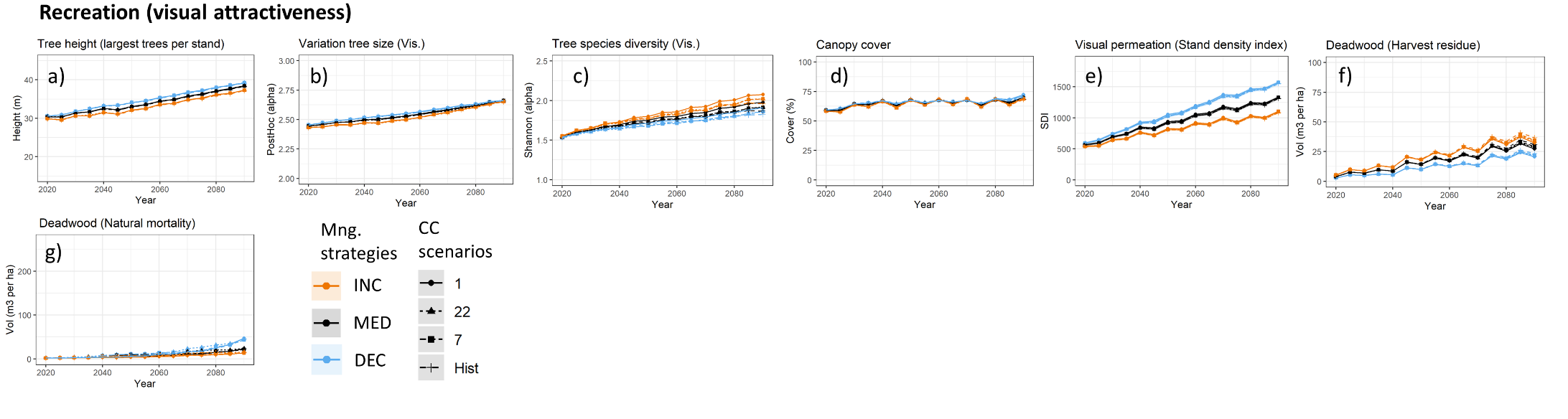


**Fig. A2.8 Indicators for recreation (visual attractiveness) under different management strategies and climate scenarios (for abbreviations, see Fig. A2.2)**


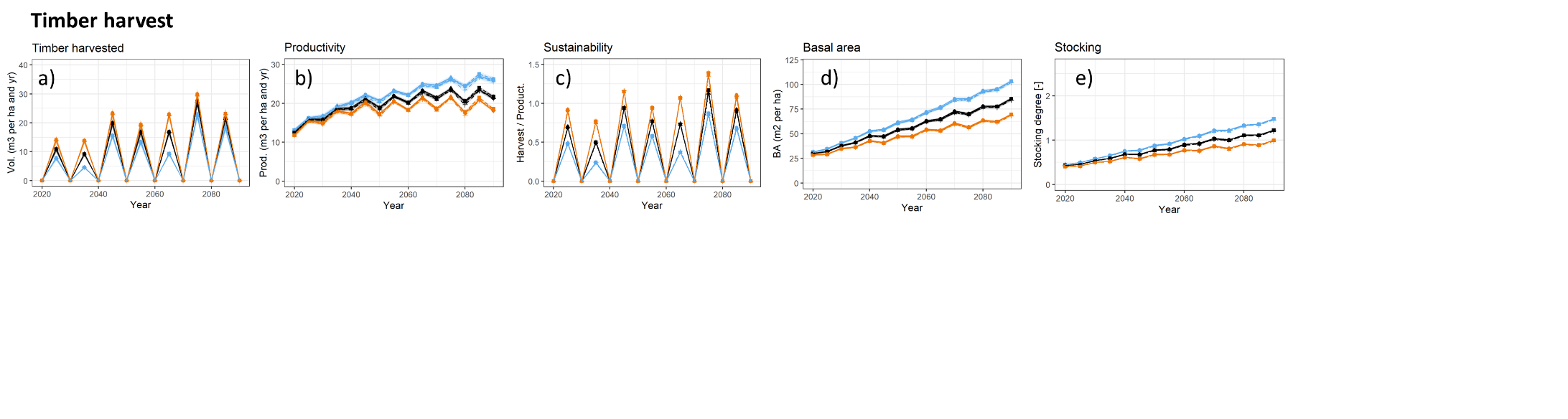


**Fig. A2.9 Indicators for timber harvest under different management strategies and climate scenarios (for abbreviations, see Fig. A2.2)**


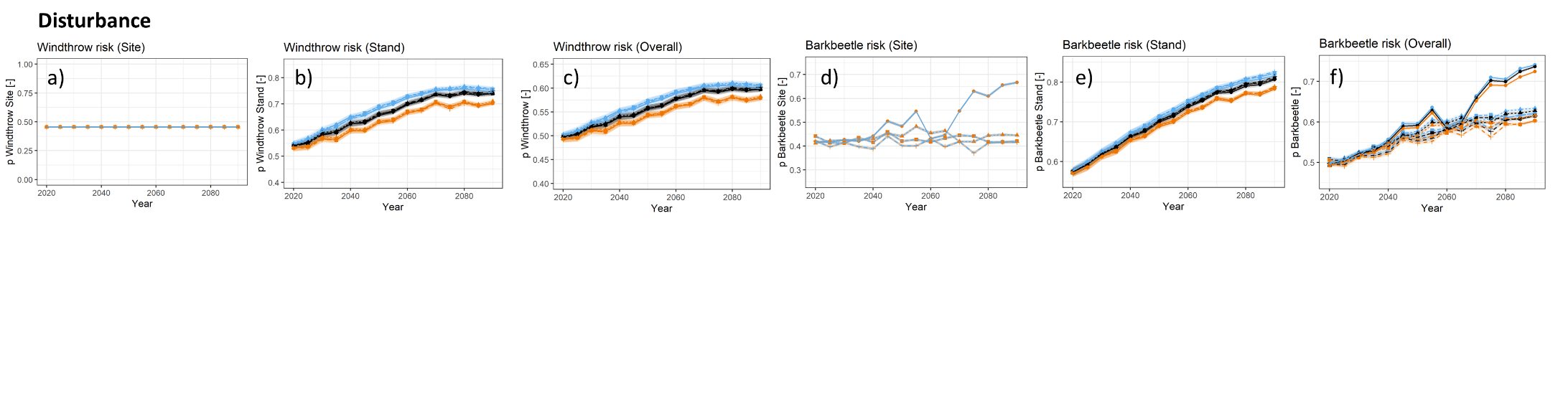
**Fig. A2.10 Indicators for disturbances under different management strategies and climate scenarios (for abbreviations, see Fig. A2.2)**

**
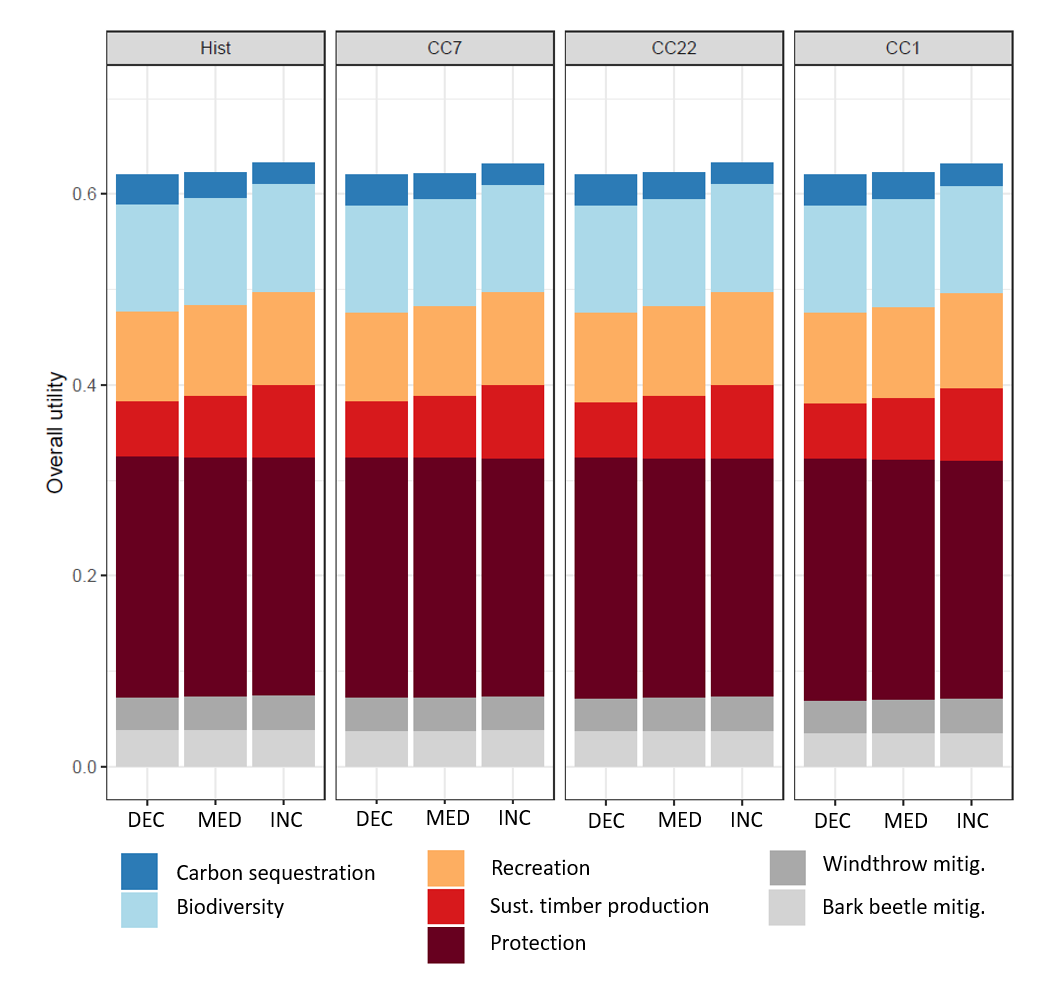
**

**Fig. A2.11 Overall utility (multifunctionality measure) for biodiversity and ecosystem services as well as disturbance risk mitigation, under different management intensities (LOW, MED, HIGH) and four climate scenarios (Hist, CC7, CC22, CC1) for end of 21^st^ century (mean overall utility for timespan 2010-2100)**
